# Evaluation of a Canadian social media platform for communicating perinatal health information during a pandemic

**DOI:** 10.1371/journal.pdig.0000802

**Published:** 2025-04-07

**Authors:** Gemma Postill, Neesha Hussain-Shamsy, Stephanie Dephoure, Alison Wong, Eliane M. Shore, Jeanette Cooper, Negin Pak, Christine Fahim, Danielle Kasperavicius, Tali Bogler

**Affiliations:** 1 Institute for Health Policy, Management and Evaluation, University of Toronto, Canada; 2 Temerty Faculty of Medicine, University of Toronto, Canada; 3 Department of Obstetrics and Gynaecology, St. Michael’s Hospital, Toronto, Canada; 4 Knowledge Translation Program, Unity Health Toronto, Toronto, Canada; 5 Department of Community and Family Medicine, St. Michael’s Hospital, Toronto, Canada; 6 Li Ka Shing Knowledge Institute, St. Michael’s Hospital, Toronto, Canada; Iran University of Medical Sciences, IRANISLAMIC REPUBLIC OF

## Abstract

Social media platforms, such as Instagram, are increasingly used as a source of health information; however, it is unclear how to effectively leverage these platforms during public health emergencies. @PandemicPregnancyGuide (PPG) was an Instagram account created by Canadian physicians to provide perinatal health information during the COVID-19 pandemic. We conducted a cross-sectional survey, and assessed Instagram analytics, to determine how and why users followed PPG and its impact on health decision-making. Respondents most valued posts explaining scientific articles in lay language and the delivery of content by medical experts. Topics of greatest interest were COVID-19 vaccination while pregnant (76%), COVID-19 infection during pregnancy (71%), and labour and delivery during the pandemic (69%). Respondents self-reported being more likely to use COVID-19 protective measures while pregnant (80%), receive COVID-19 vaccines in pregnancy (87%), and vaccinate their children against COVID-19 (58%) due to the information shared by PPG. Taken together, we demonstrate how healthcare professionals can effectively leverage social media to disseminate health information and improve uptake of public health recommendations. We recommend consideration of our findings in the development of future health-based social media platforms, particularly during public health emergencies or campaigns.

## Author summary

During public health crises, many people turn to social media for reliable health information, but it’s unclear how to use these platforms most effectively. The Instagram account @PandemicPregnancyGuide (PPG) was created by Canadian doctors to share trustworthy pregnancy-related health information during COVID-19. We surveyed PPG followers and analyzed their engagement with the account to understand (1) why people used PPG and (2) how it influenced their health decisions. Followers valued clear, expert-led explanations of scientific research, particularly on COVID-19 vaccination in pregnancy, COVID infection risks during pregnancy, and giving birth during the pandemic. Many reported that PPG made them more likely to follow COVID-19 prevention measures (public health recommendations), get vaccinated during pregnancy, and vaccinate their children. In summary, our findings show how doctors can use social media to effectively deliver accurate health information and promote uptake of public health preventative behaviors. These insights are of value to future health communication efforts.

## Background

Social media (e.g., Facebook, Instagram, X – formerly known as Twitter, and YouTube) is increasingly used to disseminate and consume health information [1]. Most Canadians (78%) regularly use social media, with higher proportions among females (81% vs. 73% males) and those of childbearing age (>95% of those 20-34 years) [2]. Social media is therefore a ripe and potentially impactful domain for perinatal and women’s health knowledge translation (KT). Indeed, social media platforms have been used for health education, awareness/de-stigmatization, and social support across a range of health conditions [3–5], including infectious diseases [6,7], chronic physical and mental conditions [8,9], and reproductive health [10].

The increased use of social media for health-related KT [11] and its ability to rapidly communicate information to a large audience highlights its potential for crisis communication in public health emergencies and campaigns. Social media use has also been observed to increase during emerging health crises and disasters as people seek information about the event and check on family and friends [12–16]. As an example, during the H7N9 virus outbreak in 2013, Twitter (now X) was used by individuals to make sense of the virus despite few posts containing actual information to help users know how to appropriately respond and adjust behaviours [16]. Likewise, during the 2015 Middle East Respiratory Syndrome coronavirus (MERS-CoV) outbreak, social media use increased public risk perception and preventive behaviors [17]. However, misinformation, information overload, and information oversimplification are known risks of social media and negatively impact knowledge [6,18]. During the COVID pandemic social media use also increased, with both positive (e.g., social support) and negative (e.g., misinformation, anxiety, and depression) effects [12,14]. Understanding how to develop high-quality social media KT accounts for effective public health promotion is pertinent.

At the onset of the COVID-19 pandemic, there were many questions and limited information about the impact of COVID-19 infection on pregnant individuals and their newborns. Simultaneously, there were disruptions in access to perinatal care services and their ancillary supports (i.e., including cancelling of perinatal classes and in-person appointments) [19]. To address the need to rapidly and effectively communicate reliable medical information during the pandemic, @PandemicPregnancyGuide (PPG), a social media platform on Instagram, was created by a team of primary and obstetrical care providers at St. Michael’s Hospital in Toronto, Canada. The purpose of PPG was to provide free, evidence-based, and accessible perinatal and women’s health information and to foster a virtual community for expectant families during the COVID-19 pandemic. The physician-led team behind PPG strategically chose Instagram as it allows lengthier posts, greater engagement, and was already being used by the target demographic (i.e., Canadian females of reproductive age). The design of PPG was patient-centered in that it focused on an identified need [19], emphasized usability of research, and improved with user feedback [20]. The account rapidly grew to over 43,500 followers by December 2022, demonstrating the potential need and value of this online health resource.

Relatively little is known about the elements of health-focused social media accounts, including PPG, that facilitated successful KT and potentially impacted health behaviors, particularly during times when rapid and clear health communication at a national level is needed. Analysis of the key elements that facilitate perinatal KT during health emergencies can enable development of an evidence-based framework for future health-focused social media accounts. From prior research in the 2014 Ebola epidemic, Instagram posts elicited greater engagement than those on Twitter (now X), potentially because the posts all include visuals and are lengthier [21]. For perinatal health accounts, community support is a highly valued element of health KT [10]. Whether these features promote positive perinatal health behaviour during a pandemic is unknown. Thus, our objectives were to (1) describe where PPG followers found general and perinatal health information before and during the pandemic, (2) determine the extent to which users perceived engagement with PPG to have impacted health decision-making during the pandemic, and (3) describe the elements of PPG most helpful and why.

## Methods

### Ethics statement

Ethics approval was obtained from St Michael’s Hospital Research Ethics Board (REB #22-235). Formal written consent was obtained from participants prior to their engagement in the following research activities.

### Study design

A cross-sectional, open electronic survey (e-survey) was conducted among current and past followers of PPG between April 25, 2023 and June 13, 2023. Reporting follows the Checklist for Reporting Results of Internet e-Surveys (SI CHERRIES Checklist) [22].

### Respondents

Eligible respondents were individuals aged 18 years or older who were current or past followers of PPG, proficient in English (i.e., able to provide informed consent and complete the survey) and had Internet access to complete the survey. Respondents were recruited through (1) Instagram-based stories and posts on PPG and (2) email follow-up of consenting respondents from our previous study on key concerns of pregnant individuals during the pandemic [19]. Recruitment materials were developed by the study team and are available in S1 Text. Respondents gave informed consent digitally to participate. Those who completed the survey could enter their contact information (stored separately from study data) into a draw to win 1 of 5 $25 gift cards.

### Data collection and storage

The e-survey (S1 Text) was developed and administered through Research Electronic Data Capture (REDCap), a secure Web application for building and managing online surveys and databases [23,24], with collected data housed on secure servers located at St Michael’s Hospital in Toronto, Canada. Data were only accessible to authorized individuals on the study team.

The e-survey took 15-20 minutes to complete and was divided into five sections with a total of 83 questions (counting matrix questions as one question), distributed over 8 webpages, with 1 additional page to provide contact information for the gift card draw and to optionally agree to be contacted for related future research. Adaptive questioning (i.e., questions conditionally displayed based on responses to other items) was used such that each respondent would not have to answer every question, as only applicable questions would be presented. A back button allowed respondents to review and change answers prior to submitting. Usability and technical functionality were piloted with five individuals known to the study investigators, and who met study eligibility criteria.

### Study measurements

#### Survey data.

To achieve objective 1, we asked respondents where they sought different types of health-related information before (i.e., prior to March 2020) and during (i.e., since March 2020) the COVID-19 pandemic. We also administered the Electronic Health Literacy Scale (eHEALS), an 8-item validated scale to evaluate individuals’ perceived knowledge, comfort, and skills at finding, evaluating, and applying online information to health problems [25]. Each item in the eHEALS is scored on a 5-point Likert Scale, then summed together, with higher scores indicating greater self-perception of electronic health literacy. The eHEALS score has shown psychometric validity among social media users, with a mean (standard deviation [SD]) reported eHEALS score of 30.7 (SD: 5) [26].

For objective 2, we presented respondents with screenshots of PPG content (e.g., posts, post captions, Instagram Live sessions, recorded videos, and Instagram stories) and inquired, using 5-point Likert Scales, about the extent to which each format was helpful for learning new health information. We elicited preferences for viewing content, overall trustworthiness, and general usefulness of PPG. We presented an example of a #MedicalMonday post (i.e., a weekly post that summarized a recent research article in lay language, often related to COVID-19 infection or vaccination), and asked respondents, using a 5-point Likert Scale, about their ability to understand the science content (e.g., how well respondents understood the content), preferences around study methods, whether they only viewed takeaway points, and whether the health information was helpful. We inquired about accessibility of content using closed-ended survey questions. The readability of each post was separately assessed using the Flesch Reading Ease score and Flesch-Kincaid Grade Level [27,28]. Respondents reported their timeline of following PPG, when they utilized PPG most, and the topics of highest interest during those time periods. Respondents were also provided with an opportunity to use free text responses to supplement or expand on their responses when appropriate.

For objective 3, we asked respondents about the impact of PPG on COVID-19 protective behaviors (e.g., masking and distancing) while pregnant or around vulnerable populations, receiving a COVID-19 vaccine while pregnant, trying to conceive, or breastfeeding, encouraging others (e.g., social circle and children) to get a COVID-19 vaccine, and breastfeeding while having an active COVID-19 infection, all common topics of misinformation at the time [29].

Sociodemographic data (e.g., age, gender, sexual orientation, race/ethnicity, income, education, location of residence, employment, and parental leave status) were collected at the conclusion of the e-survey. All survey questions were optional to complete.

#### Social media usage data.

We accessed Instagram data analytics (Insights) for PPG usage between April 2020 and July 2023. Insights provide information on trends related to followers and engagement with posts, videos, stories and Instagram Lives, with information provided at an aggregate level, not individual level [30]. Metrics assessed included accounts reached (“number of unique accounts that have seen your content on screen at least once”), accounts engaged (“number of unique accounts that have interacted with your content”), and content interactions (“actions people take when they engage with your content, such as likes, comments, saves, shares, and replies”) for individual posts, videos, stories, and Instagram Lives [30].

### Analysis

We calculated the view rate (ratio of survey visitors/site visitors), participation rate (ratio of visitors who agreed to participate/visitors to the first survey page), and completion rate (ratio of users who finished the survey/users who agreed to participate) using the number of individual responses in REDCap. Those who completed at least 80% of the survey were included in the analysis. The IP addresses of individual users were not collected; therefore, calculations were made with the assumption that each survey visitor was a unique individual. Timestamps of surveys were evaluated to remove entries submitted after the closure of the survey.

We used a descriptive analysis for the quantitative survey data. The analysis was done using R version 4.0.3 to calculate response frequency [31].

Open-ended (i.e., free text) survey data were analyzed using qualitative content analysis [32]. Two researchers (JC and NP) reviewed the free text responses to produce an initial codebook. Using the codebook, researchers (JC and NP) double coded 20% of responses for the open-ended survey questions. Once double coding was complete a consensus discussion was conducted. During the consensus discussion, the two researchers compared coding, discussed discrepancies, and finalized code definitions to ensure codes were clear and similarly used by both researchers. After the consensus discussion, remaining responses were divided and single coded by one of the two researchers using the finalized codebook. The coded data were then used to summarize the main themes of the free text survey responses (S1 Codebook). In creating the codes, in vivo labels of themes (i.e., direct respondent quotes) were used where possible.

## Results

### Study sample

**Fig 1:**
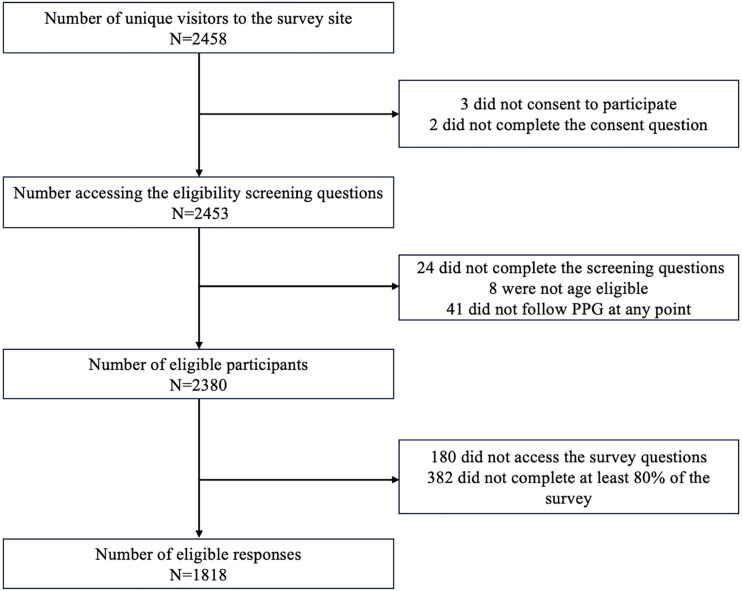
Diagram of Included Respondents. Diagram of survey respondents included in analysis. The survey view rate (e.g., number who viewed the survey on the site after clicking the link) was 100% (N=2453/2453), the participation rate was 99.8% (N=2453/2458), and the completion rate was 74.1% (N=1818/2453).

Overall, 2458 individuals clicked the link from recruitment materials and were directed to the survey (Fig 1). The survey had a participation rate of >99% (n=2453) and a completion rate of 74% (n=1818). In total, 1818 survey responses were included for analysis (Fig 1).

Respondents’ mean age was 36 years (SD: 4, range: 24 to 70) (Table 1). Most respondents (n=1740, 95%) resided in Canada, had an annual household income greater than $120,000 CAD (n=1256, 69%), were university educated (n=1620, 89%), and identified as heterosexual (n=1673, 96%) and/or white (n=1567, 86%). The mean eHEALS score across respondents was 28.6 (SD: 5). On average, respondents had previously had 2 pregnancies (SD: 1), with 94% of respondents (n=1717) having at least one child born during the pandemic.

**Table 1:** Characteristics of study sample. *Sociodemographic data as self-reported by survey respondents (N=1818). Data are presented as n (%) unless otherwise specified.*

	n (%)	
**Age**, mean (SD)	35.96	(3.75)
**Woman** (n=1817)	1805	(99.3)
**Sexual orientation** (n=1749)		
Heterosexual	1673	(95.7)
Gay, Lesbian, Queer, Bisexual, Pansexual	63	(3.6)
Others	13	(0.7)
**Ethnicity**		
White	1567	(86.2)
South or Southeast Asian	106	(5.8)
East Asian	88	(4.8)
Indigenous	35	(1.9)
Black	27	(1.5)
Others	106	(5.8)
**Education level**		
Graduate or professional degree	913	(50.2)
Bachelor’s degree	707	(38.9)
Post-secondary education or training	175	(9.6)
High school or less	18	(1.0)
Prefer not to say	5	(0.3)
**eHEALS score**, mean (SD)	28.6	(5.4)
**Employment status** (N=1815)		
Full-time	1089	(60.0)
Part-time	154	(8.5)
On leave	399	(22.0)
*On leave: Parental Leave*	*386*	*(96.7)*
*On leave: Medical, disability, other, or prefer not to say*	*13*	*(3.3)*
Stay-at-home parent	118	(6.5)
Unemployed	9	(0.5)
Student	11	(0.6)
Other or prefer not to say	35	(1.9)
**Household income** (N=1815)		
<$59,999 CAD	44	(2.4)
$60,000-$89,999 CAD	115	(6.3)
$90,000-$119,999 CAD	262	(14.4)
>$120,000 CAD	1256	(69.2)
Do not know or prefer not to say	131	(7.2)
**Country of residence** (N=1812) ^2^		
Canada	1740	(96.0)
*Canada: Maritimes (NF, NS, NB, PEI)*	*49*	*(2.8)*
*Canada: Quebec*	*29*	*(1.7)*
*Canada: Ontario*	*1448*	*(83.2)*
*Canada: Prairies (MB, SK)*	*55*	*(3.2)*
*Canada: Western Canada & Territories* *(AB, BC, YK, NWT, NV)*	*156*	*(9.1)*
United States	53	(2.9)
Other	19	(1.0)
**Area of residence** (N=1800)		
Urban	1164	(64.7)
Semi-urban	426	(23.7)
Rural	204	(11.3)
**Health status** (N=1812)		
Very good	543	(30.0)
Good	1110	(61.2)
Fair, poor or very poor	159	(8.8)
**Current reproductive status** (N=1808)		
Postpartum	789	(43.6)
Currently pregnant	250	(13.8)
Trying to conceive	190	(10.5)
None of the above	579	(32.0)
**3**^+^ **doses of COVID-19 vaccine received,** (N=1783)	1628	(91.3)
**Reproductive status during 1**^**st**^ **and 2**^**nd**^ **COVID Vaccine Dose** (N=1792)		
Thinking of or trying to get pregnant	336	(18.8)
Pregnant	623	(34.8)
Breastfeeding	630	(35.2)
None of the above	203	(11.3)
**Reproductive status during 3**^**rd**^ **and/or 4**^**th**^ **COVID Vaccine Dose** (N=1792)		
Thinking of or trying to get pregnant	178	(9.9)
Pregnant	475	(26.5)
Breastfeeding	633	(35.3)
None of the above	345	(19.3)
Not Applicable	142	(7.9)
**Number of pregnancies**, mean (SD)	2.24	(1.21)
**Number of live births**, mean (SD)	1.56	(0.74)
**Number of household members**, mean (SD)	3.53	(0.86)
**Number of children in household**, mean (SD)	1.58	(0.76)
**Child born during the pandemic** (n=1818)	1717	(94.4)

1 Reported as n (%) with N=1818 unless otherwise specified (unless otherwise specified).

2 N=1737 respondents reported their province of residence; this number is used to calculate percent.

Most respondents received 3 or more doses of the COVID-19 vaccine (n=1628, 91%). At the time of their initial or second COVID-19 vaccine, most (n=1589, 89%) were either pregnant, trying to conceive, or breastfeeding. At time of their third or fourth booster dose, 77% (n=1286/1650) were pregnant, trying to conceive, or breastfeeding (Table 1).

### Objective 1: Sources of health information

**Fig 2:**
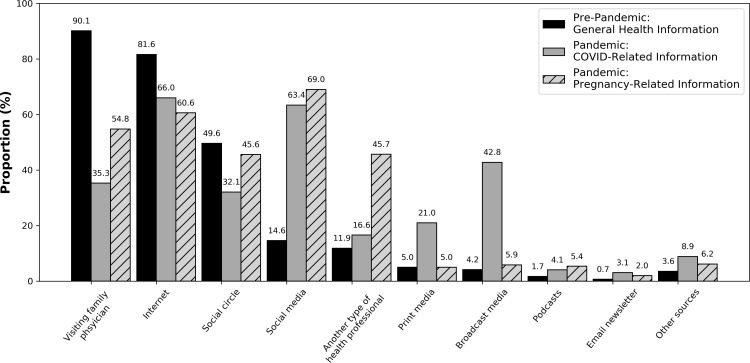
Sources of health information for pre-pandemic general health information, COVID-19 health information, and pandemic pregnancy-related health information. Sources of health information captured as self-reported by survey respondents. Complete data for this figure can be found in S1 Data.

Prior to the pandemic, the most common sources of general health information were family physicians (n=1636, 90%), the internet (n=1481, 82%), and social circles (n=901, 50%) (Fig 2). During the pandemic, sources of health information shifted toward greater use of the Internet and social media. Specifically, the most common sources of *COVID-19 information* were the Internet (e.g., Google, websites, blogs) (n=1198, 66%), social media (n=1150, 63%), and family physicians (n=641, 35%). Similarly, the most common sources of *pregnancy-related health information* during the COVID-19 pandemic were social media (n=1254, 69%), the Internet (n=1101, 61%), and family physicians (n=997, 55%) (Fig 2).

Respondents were most often referred to PPG through family/friend recommendations (n=603, 31%), Instagram recommendations (n=581, 29%), and their healthcare provider (n=232, 12%) (Table 2). Respondents reported the most frequent use of PPG near the start of the pandemic; 63% (n=915) reported following PPG between March 2020 to January 2021 (S1 Data).

**Table 2:** Social media health information use. *Use of social media platforms for health information during the pandemic among respondents (N=1818).*

	n (%)	
**Social media platforms used for health information (n=1818)**		
Instagram	1732	(95.4)
Facebook	553	(30.5)
Twitter^1^	281	(15.5)
YouTube	260	(14.3)
Reddit	227	(12.5)
Messaging apps	188	(10.4)
Others	342	(18.8)
Do not use	54	(3.0)
**How respondents discovered @PPG (n=1817)** ^**2**^		
Family/friend recommendation	603	(33.2)
Instagram recommendation	581	(32.0)
Healthcare provider recommendation	232	(12.8)
Do not remember	456	(25.1)
Other^3^	98	(5.4)
**Pregnancy-related health topics of greatest interest (n=1818)**		
COVID-19 vaccination while pregnant	1387	(76.3)
COVID-19 infection while pregnant	1292	(71.1)
Labour and delivery during COVID-19	1246	(68.5)
COVID-19 infection in newborns/children	1174	(64.6)
COVID-19 vaccination while breastfeeding	1060	(58.3)
COVID-19 infection while breastfeeding	799	(43.9)
COVID-19 preventative measures	752	(41.4)
COVID-19-19 vaccination in children	738	(40.6)
Pregnancy and postpartum topics	648	(35.6)
COVID variants	633	(34.8)
Impact of COVID-19 on child development	425	(23.4)
Infant and child health (general)	414	(22.8)
Labour and delivery (general)	396	(21.8)
Mental health	356	(19.6)
COVID-19 vaccination while trying to conceive	313	(17.2)
General women’s and/or sexual health	282	(15.5)
COVID-19 infection while trying to conceive	199	(10.9)
Personal wellness	164	(9.0)
Other	11	(0.6)

^1^Now called X.

^2^Question was formatted as a choose all that apply; numbers do not add to 100%.

^3^Respondent reporting other specified: Other Instagram account recommendation (n=39), News Article (n=19), Post on other social media (n=16), Television star (n=9), Doctor recommendation (n=8), Social circle recommendation (n=6)

### Objective 2: Impact of PPG on health-decision making

**Fig 3:**
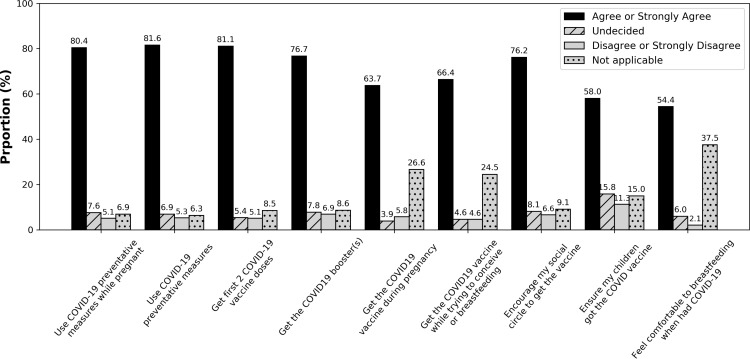
Influence of PPG on Self-Reported Behaviours of Respondents (N =1818). Self-reported behaviour changes as a result of information shared by PPG. Responses address the question “Because of PPG I was more likely to…” (see S1 Survey).

Participants reported that PPG made them more likely to use COVID-19 protective measures when pregnant (n=1454, 80%), use protective measures around those not eligible for vaccination or more vulnerable (n=1473, 82%), and encourage their social circle to pursue vaccination (n=1374, 76%) (Fig 3, S1 Data).

For most respondents, information shared by PPG influenced their likelihood to get vaccinated against COVID-19 in pregnancy (n=1150, 87%) or while trying to conceive or breastfeed (n=1194, 88%, Fig 3 and S1 Data). In general (e.g., both during and outside of pregnancy), 81% (n=1467) were more likely to get the first two doses and 77% (n=1383) were more likely to get a third dose because of PPG. As well, 58% (n=1045) of respondents reported being more likely to have their children receive COVID-19 vaccines because of information shared by PPG (Fig 3 and S1 Data).

### Objective 3: Most helpful elements of PPG

#### Reliable source of information.

Most respondents felt that PPG made it easier to understand health information (n=1769, 98%), was a reliable source of information (n=1786, 99%), shared truthful information (n=1786, 99%), could be trusted not to share or spread misinformation (n=1779, 99%), and helped dispel misinformation from other sources (n=1660, 92%) (S1 Data).

Specific to COVID-19 information, most respondents felt that PPG allowed them to stay up to date on research related to COVID-19 and perinatal health (n=1788, 99%) as well as public health policies (n=1764, 98%) (S1 Data). Respondents felt that information shared by PPG could not be obtained from their healthcare provider (n=1246, 69%) (S1 Data). Notably, respondents reported that it was important to them that PPG was run by physicians (n=1713, 95%) and provided Canadian-specific content (n=1693, 94%) (Table 3).

**Table 3:** Helpfulness of PPG features. *Helpfulness of PPG account features as self-reported by respondents (N=1818). Data are reported as n (%) unless otherwise specified.*

	n (%)	
**Posts Explaining Scientific Studies** (n=1814)		
Helpful	1805	(99.5)
Neural or unhelpful	1 – 5	(≤0.3)
Not applicable	0	(0.0)
**Captions of PPG posts** (n =1816)		
Helpful	1691	(93.1)
Neural or unhelpful	1 – 5	(≤0.3)
Not applicable	7	(0.4)
**Instagram live sessions** (n=1817)		
Helpful	1148	(63.2)
Neural or unhelpful	1 – 5	(≤0.3)
Not applicable	430	(23.7)
**Recorded videos** (n=1816)		
Helpful	1223	(67.3)
Neural or unhelpful	1 – 5	(≤0.3)
Not applicable	396	(21.8)
**Instagram stories** (n=1817)		
Helpful	1624	(89.4)
Neural or unhelpful	0	(0.0)
Not applicable	94	(5.2)
**Account being led by physicians** (n=1812)		
Important	1713	(94.5)
Not Important	99	(5.5)
**Canadian specific content** (n=1810)		
Important	1693	(93.5)
Not Important	117	(6.5)
**Community support** (n=1813)		
Important	865	(47.7)
Not Important	948	(52.3)

Respondents also felt that the PPG community helped them feel less alone during the pandemic (strongly agree: n=943, 52%), and less worried or anxious in their pregnancy journey (strongly agree: n=832, 46%).

#### Preferred format and accessibility of content.

Almost all (n=1805, 99.5%) respondents found posts, specifically those summarizing scientific studies, to be the most helpful format and 93% (n=1691) reported captions (i.e., text alongside posts) to be helpful as well. Instagram analytics revealed #MedicalMonday posts (posts summarizing research studies) to have the most overall engagement (measured by likes, comments, saves, and shares). Other valued formats for content delivery were Instagram stories (n=1624, 89%), recorded videos (n=1223, 67%), and live sessions (n=1148, 63%) (Table 3).

Most respondents found content understandable (n=1764, 97%); the mean Flesch Reading Ease score for #MedicalMonday posts was 16.7 (SD: 12) and the mean Flesch-Kincaid Grade Level was 20.9 (SD: 6) (S1 Data). Only 5% (n=88) reported too much science language within posts (S1 Data).

#### Content with greatest engagement.

During the periods of greatest reliance on PPG, self-reported topics of greatest interest were COVID-19 vaccination while pregnant (n=1387, 76%), COVID-19 infection during pregnancy (n=1292, n=71%), and labour and delivery during the COVID-19 pandemic (n=1246, 69%) (Table 2). Similarly, based on Instagram Insights, the post with most shares to other Instagram users was a #MedicalMonday post on COVID-19 antibodies in infants following maternal vaccination in pregnancy (posted February 2022, 1523 shares, Flesch-Kincaid Grade Level = 30.5, S1 Data).

### Qualitative analysis

The nine qualitative themes identified were: (1) *trustworthy and reliable*, referring to participants’ confidence in PPG as a dependable source of health information; (2) *evidence-based*, emphasizing the importance of scientifically supported content; (3) *easy to understand*, highlighting the accessibility of language and clarity of information; (4) *run by trusted professionals*, denoting the added credibility and user confidence in content creators; (5) *up-to-date and relevant*, ensuring that the content remained applicable to users’ needs; (6) *locally-based and Canadian*, making the information more contextually relevant; (7) *easy to access*, stemming from reduced barriers to engage with content; (8) *provided perinatal and children’s health information*, addressing key areas of interest for the target audience; and (9) *community support*, owing to the sense of connection and encouragement among users (Table 4 and S1 Codebook). Together, these themes provide further understanding of why PPG was perceived to be helpful and encouraged health promoting behaviours, supporting the quantitative insights.

**Table 4:** Qualitative themes identified in free-text responses.

Qualitative Theme and Definition	Exemplar Quote
**Trustworthy and reliable.** PPG was perceived as a dependable source of accurate and credible information about pregnancy and COVID-19, fostering confidence among its audience.	*“I like that I can trust the information being presented. It truly helped me feel less worried/anxious and more informed while pregnant when the pandemic started. I found it extremely helpful after giving birth as well as for general information about the vaccine and also specific info about the vaccine while breastfeeding. –* P467
**Evidence-based.** Participants valued that PPG provided information grounded in scientific research and evidence, reinforcing their trust in the guidance offered. The linking of content to scientific sources also enabled the sharing and verification of information.	“*I liked that the posts were all cited, and research based as well. PPG also helped me decided to get my baby vaccinated when she was old enough, again thanks to the clear and research backed information*” – P449
**Easy to understand**. PPG effectively communicated complex medical information in a clear and comprehensible manner, using a familiar platform to enhance accessibility. They also describe PPG as making primary research results more accessible to a wide audience.	*“Easily accessible information that I can trust, breaks down medical information so it is easy to understand and directly applicable to my daily life.” –* P187
**Run by trusted professionals.** The credibility of PPG was strengthened by its association with clinicians actively caring for pregnant and postpartum patients. Additionally, the representation of women and mothers among the professionals was noted as meaningful.	“[I followed information produced by PPG because it was] *an account created by doctors that is as credible as possible. It’s packaged up and well formatted for busy people wanting to get the most important information.”* – P35
**Up-to-date and relevant.** Participants appreciated PPG’s timely dissemination of current and pertinent information, often addressing emerging concerns before traditional healthcare channels. Several participants mentioned that PPG was one of their “go-to” resources because they could often find information about new research before other sources, such as their doctors, knew about it.	*“I love that PPG is succinct, clear, and presents information with a balanced approach. Feeling like I had an up-to-date, reliable, resource that was available to me during an incredibly stressful time, was imperative in helping me mediate my anxiety during my pregnancy and postpartum. The information on PPG made me feel confident in advocating for an early vaccination while pregnant. I got vaccinated at the earliest possible opportunity – 2 weeks prior to my delivery date”. –* P95
**Locally-based and Canadian.** Participants noted that it was important to them that PPG was run by local physicians (their hospital, city, province, or country) because they knew information about things that varied across jurisdictions (e.g., public health restrictions) was relevant for them.	*“While pregnant at the height of COVID, I relied heavily on the information and community that PPG gave me. I felt an overwhelming sense of calm and reassurance hearing from real Canadian doctors when I could barely get through to my own physician.” –* P584
**Easy to access.** Participants highlighted the convenience of accessing PPG content through familiar platforms, as well as enabling access to primary research that they would not otherwise come across in their routine use of social media.	“[The content was] *easy to access, read & share. Easy to verify accuracy & reputation of information. I liked just seeing things in my feed. Sometimes I would come across information I’d file away for later*.” – P8
**Provided perinatal and children’s health information.** PPG addressed critical gaps in pandemic-related health information specific to pregnancy, postpartum care, and children’s health. Participants denoted the importance of having a resource that provided information specific to pregnancy, postpartum, children, and women’s health during the pandemic.	“[I valued that content was] *backed by research, physician led, info during pandemic specific to pregnancy and young children which was severely lacking from government/other news outlets*.” – P645
**Community support.** Participants valued the supportive and non-judgmental tone of the PPG community, describing it as a space for connection, shared experiences, and emotional reassurance. Some also appreciated the support from knowing other followers were in similar situations.	“[PPG] *felt like a community. A place to ask questions when health care systems were overburdened*.” – P1424

## Discussion

Our study identifies (1) where @PandemicPregnancyGuide (PPG) followers found general and perinatal health information before and during the pandemic, (2) the elements of PPG that were most helpful, and (3) the extent to which engagement with PPG impacted health decision-making during the pandemic. We identified a shift among respondents in sources of health information from family physicians prior to the pandemic to social media and the Internet during the pandemic. Respondents valued PPG for providing reliable and trustworthy medical information in an easy-to-understand format, which ultimately influenced the uptake of COVID-19 protective measures, including vaccination for themselves and family members. Taken together, our findings highlight how social media can be an important source of health information during a public health emergency or campaign. These results can be used to guide the development of an evidence-based framework for future health-focused social media accounts.

### Use of social media in public health emergencies

Similar to @PandemicPregnancyGuide, social media was used by institutions and individuals for crisis communication during the COVID-19 pandemic [33–36]. We highlight how social media became respondents’ primary source of health information during the pandemic with a coinciding relative decrease in other information sources (e.g., primary care providers and family members); the shift from other sources to social media owing to accessibility has been noted elsewhere in the literature [11,37,38]. Family physicians remain a trusted, valuable source of health information [39–41]. Our finding of a shift to social media and of PPG as a trusted source of health information may be due to the account being run by primary care providers on a platform that individuals were already using. Together, these findings suggest that healthcare providers should be aware of, potentially engage with, and encourage the use of reputable social media accounts during public health emergencies.

### Elements of successful knowledge translation platforms

Prior to our study, limited literature explored the elements of social media KT that drive success in public health emergencies, particularly for vulnerable populations such as pregnant individuals. In health-related KT, how messages are framed, who delivers the message, and how users can engage with one another on the platform, are critical for positively affecting vaccine uptake [42]. To inform future KT of medical information on social media, we identified elements that drove PPG’s success: delivery of content by local medical professionals and posts that succinctly summarize up-to-date scientific information in lay-language. Trust in medical professionals [43] and their ability to dispel misinformation during crises have been observed elsewhere in the pandemic literature [13,34,44,45]. Respondents in our study highlighted the importance of receiving scientific information in lay language (e.g., #MedicalMonday posts), which, despite moderate-difficult language level, including medical language, were thoroughly and sufficiently explained in detail such that respondents understood the content. Note, our respondents were mostly young, highly educated women, as reflected by their mean eHEALS score, which is reflective of the social media population reported elsewhere [26]. Our respondents’ desire for scientific information suggests that during public health crises, the public may seek out more detailed information and knowledge to fully understand the situation and guide their response.

### Impact on self-reported health-behaviour change

Importantly, the impact of PPG extended beyond knowledge dissemination to self-reported positive health-behaviour change (e.g., masking, vaccination, breastfeeding, and other COVID-19 prevention measures), highlighting the utility of effective social media KT for these types of health-behaviour change. In other areas of medicine, social media has also shown utility for positive health-behaviour change [46–48]. For public health promotion and in pandemics, common frameworks for conceptualizing the relationship between social media and other public health campaigns and health promotion behaviour include the Health Belief Model [49], the Social Cognitive Theory [50–54], as well as the Theoretical Domains Framework [55], among others. The Health Belief Model (HBM) posits that six constructs predict health behavior: risk susceptibility, risk severity, benefits to action, barriers to action, self-efficacy, and cues to action [56].The Social Cognitive Theory denotes the improved adoption of new behaviour when it is observed in others.[53] The Theoretical Domains Framework provides a systematic, structured approach for integrating multiple well-supported theories on health behaviour (including the Health Belief Model and the Social Cognitive Theory).[57,58] Given the multiple components of PPG (scientific information, expert voices, community support, etc.) references by our participants as encouraging their adherence to guidelines, we believe our findings support the Theoretical Domains Framework. However, the impact of knowledge disseminated by PPG and its community versus that of other confounding variables (higher education, “wait-and-see what others do” action cue from physicians, accessibility, etc.) cannot be distinguished in our study [59]. Regardless of the mechanism, respondents stated being more likely to engage in positive preventative health behaviours because of PPG.

For vaccination, our respondents reported increased likelihood of receiving a COVID-19 vaccine (in general, during pregnancy, and while trying to conceive or breastfeeding), vaccinating their children, and encouraging their social circle to get vaccinated. Our respondents attributed this to the knowledge shared by PPG. The greatest barriers to vaccine uptake among pregnant individuals have been reported as misinformation and safety concerns, particularly fetal/child development [60,61]. A meta-analysis found a positive correlation between knowledge about the COVID-19 vaccine and vaccine acceptance rates among pregnant individuals [62]. The relationship between knowledge and vaccine acceptability may partially explain why 89% of our sample received their initial COVID-19 vaccines while pregnant, trying to conceive or breastfeeding, compared to 49% in the pregnant population globally [62].

### Use of social media in public health emergencies

Taken together, our findings demonstrate how social media, specifically Instagram, can be used for KT and health promotion in future public health emergencies. Our platform was effective in promoting uptake of public health guidelines and highly valued by users. We present five key take-away messages for health practitioners or organizations developing health-focused social media KT platforms: (1) choose a social media platform based on target-audience and communication needs; (2) leverage medical professionals and expert voices; (3) focus on explaining scientific evidence in lay terms; (4) provide up-to-date and relevant information (e.g., identifying areas of concern via regular callouts and engagement with followers; and (5) foster trust in the information provided. Practitioners also need to both create and manage accounts. PPG was not able to continue post-pandemic due to the non-compensated human resource burden. Given the value of such platforms and the resources needed to maintain it, we propose governments and public health organizations invest in and leverage clinician-informed social media accounts as an avenue for KT, while considering the human and financial resources needed to ensure sustainability and adaptability.

### Limitations and future work

Our study is not without limitations. First, our study was not conducted during peak PPG usage and thus, our sample represents a small proportion (6%) of PPG’s total followers, potentially introducing non-response bias. However, this study was only feasible to conduct when the crisis of the pandemic subsided. Second, we were unable to verify whether multiple survey completions occurred as we did not collect IP addresses. Third, by focusing on followers, the study captured a group already inclined to seek and trust medical information from social media sources—particularly this specific account. Our findings are not reflective of non-followers, individuals who consciously avoid health-focused accounts, and users of other social media platforms. Fourth, there is discrepancy between objective and subjective assessment of posts’ accessibility. We used the Flesch Reading Ease score to measure the difficulty of our posts (which were of higher reading comprehension than the average Canadian population); however, this tool does not capture the context of words (e.g., if followed by understandable definitions and explanations) nor the increased public understanding of words previously considered jargon (e.g., *contact-tracing* and *quarantine*). Thus, it is possible that our posts were more understandable than the grade level tool reflects. This combined with the higher education level of respondents, may explain why most respondents reported PPG content as easy to understand [63,64].

Fifth, our data on health behaviour change was retrospective and self-reported; therefore, our findings may be subject to self-reporting, social desirability, and recall biases [65]. Given our objectives (the *perceived* impact of PPG on health behaviours), this approach was appropriate. Previous literature has also demonstrated positive associations between individuals’ *perception* of health events and behavioural change, and the connection between self-efficacy and *actual* health behaviour change [66,67]. Future research can mitigate the possible influence of recall and social desirability biases through collecting objective measures of adherence over time (e.g., pre- and post-vaccination records). Additionally, in describing platform effectiveness, we used a descriptive (vs. causal) approach and did not distinguish between types of users (those expectant, new parents, healthcare providers, etc.) in the descriptive analysis given the limited sample size. While many respondents attributed health decisions (e.g., masking and vaccination) to PPG, other influences, such as public health campaigns or physician advice, were not accounted for and may confound this relationship. Lastly, our results may not generalize to different socioeconomic and cultural groups, nor to those not using social media. Our study sample was skewed towards higher income and education levels and was predominantly white. Our findings thus have limited applicability to less-educated or less-digitally literate populations, who may also find the content not as easy to understand. However, our sample is reflective of the demographic using Instagram [68]. Our sample demographics precluded our ability to conduct sub-group analyses in order to determine differences in the use of PPG among different demographic groups.

Despite these limitations, we demonstrate that PPG was a valuable and reliable source of up-to-date perinatal-related information during a public health emergency. Our account was focused on a health topic (pregnancy), providing comprehensive information pertaining to misinformation, new studies, and public health policy; such approach was effective, and we recommend its use in future public health emergencies. With the shift in users turning to social media and the internet for health information, it is important for physicians to be aware of, potentially recommend, and engage with reputable health-based social media accounts to further bolster these accounts and dispel misinformation. Further evaluation should explore the effect of social media health platforms across subgroups. Likewise, additional research is needed to understand the long-term use and impact of PPG and similar accounts after the health crises have subsided; it is possible that those who followed PPG continue to have higher acceptance rates for maternal and child immunization.

## Conclusion

Our study highlights key components of a successful social media platform for maternal and child health KT during a public health emergency: leveraging medical professionals, focusing on scientific evidence, providing up-to-date and relevant information, engaging expert voices, and fostering trust in the information communicated. Future health-based KT platforms should incorporate these elements. We also demonstrate the ability of a social media platform to influence positive health-behaviours among pregnant individuals and have positive impacts that extend beyond the primary user (to their social circles and family members). Followers reported being more likely to use public health protective measures, receive COVID-19 vaccines during pregnancy, and encourage others to do the same. Together, these results provide convincing evidence for the use of social media for KT and for influencing positive health-behavior change during a health crisis. Future KT platforms and research evaluations must consider equity implications, including comfort with and access to digital platforms, to ensure that all individuals have up-to-date health information in public health emergencies.

## Supporting information

S1 Checklist CHERRIES Checklist.
Study reporting guidelines.
(DOCX)

S1 Text Study recruitment material. (DOCX)

S1 Survey Study survey distributed to participants. (PDF)

S1 Data Additional quantitative survey data. (DOCX)

S1 Codebook Qualitative codebook derived from open-ended survey responses. (DOCX)
