# Supplementary material for: Evaluation of a Canadian social media platform for communicating perinatal health information during a pandemic
Source: PLOS Digit Health. 2025 Apr 7;4(4):e0000802. doi: 10.1371/journal.pdig.0000802 (PMC11975109; doi:10.1371/journal.pdig.0000802)
Supplement: S1 Text — (DOCX) [file pdig.0000802.s002.docx]

### Recruitment Materials

Recruitment for the study will occur through two means:

1. An email will be sent to the consenting participants of a previous study on the @PandemicPregnancyGuide (**REB 20-116:** @PandemicPregnancyGuide Study: A cross-sectional survey given via social media to understand sources and level of distress among pregnant individuals during the COVID-19 pandemic) and
2. The following are posts will be made on @PandemicPregnancyGuide for recruitment to the study

Below is the messaging that will be used to recruit participants, which we have tailored to the two forementioned audiences.

**Content of the Email Recruitment to Previous Study Participants**

Dear [Participant’s name],

You are receiving this email because you were a participant in a study related to the Instagram account @PandemicPregnancyGuide (**REB 20-116:** @PandemicPregnancyGuide Study: A cross-sectional survey given via social media to understand sources and level of distress among pregnant individuals during the COVID-19 pandemic) and consented to be contacted regarding further research related to @PandemicPregnancyGuide.

We are interested in learning how and why individuals used @PandemicPregnancyGuide as a health information resource during the COVID-19 pandemic by launching a research study.

Participation in the study involves completing a voluntary survey (approximately 20 minutes) and with the information you share, we are hoping to better understand how @PandemicPregnancyGuide can improve its outreach and continue to create content on health topics that are useful and empowering for its users.

Through completing the survey, you will also be able to be entered into a chance to win 1 of 10 $25 gift cards.

For more information and to sign up, please visit [URL] or contact Dr. Tali Bogler at the [pandemicsandpregnancy@gmail.com](mailto:pandemicsandpregnancy@gmail.com)

**Content of the**  **Social Media Post (Posted on the @PandemicPregnancyGuide Account)**

Participants needed for a study on @PandemicPregnancyGuide!

Did you or do you follow @PandemicPregnancyGuide?

We are interested in learning about how and why you used PPG as a health information resource during the COVID-19 pandemic and how you will use it post-pandemic.

You may be eligible if you…

- Are aged 18 years or older
- Are comfortable completing a survey in English
- Can access the internet to complete the survey
- Are a current or previous follower of the @PandemicPregnancyGuide Instagram account

Participation involves completing a short questionnaire (estimated time 10-15 minutes).

Through completing the survey, you will also be able to be entered into a chance to win 1 of 10 $25 gift cards.

For more information and to sign up, please visit the link in our bio.
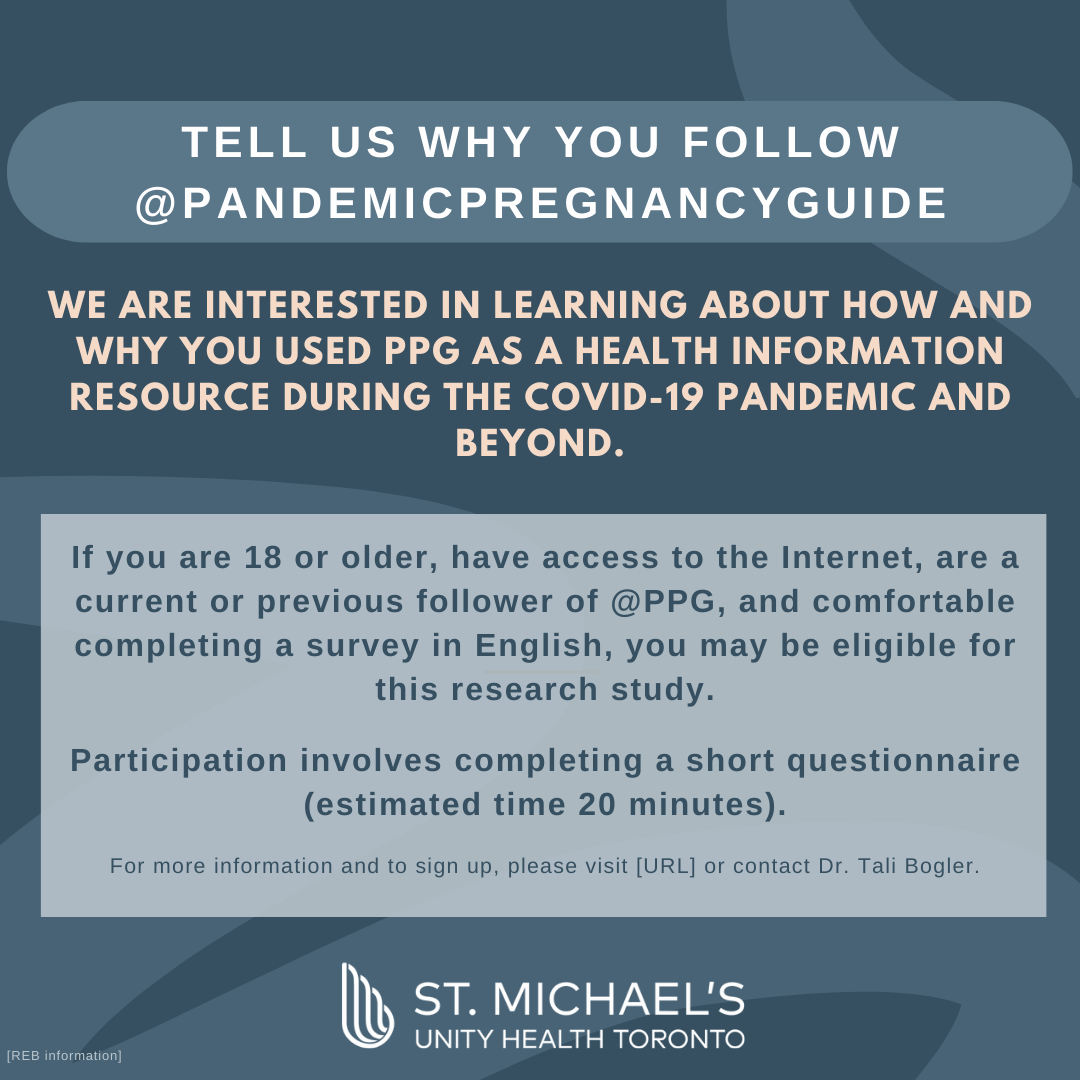


**Sample caption for posts (i.e., a text caption following an image posted directly to our Instagram account feed)**

We are interested in learning how and why individuals used @PandemicPregnancyGuide as a health information resource during the COVID-19 pandemic by launching a research study.

⠀

The voluntary survey takes approximately 10-15 minutes to complete and with the information you share, we are hoping to better understand how @PandemicPregnancyGuide can improve its outreach and continue to create content on health topics that are useful and empowering for its users.

For more information and to sign up, please visit the link in our bio.

Thank you for considering, we appreciate your time.
