# Supplementary material for: Evaluation of a Canadian social media platform for communicating perinatal health information during a pandemic
Source: PLOS Digit Health. 2025 Apr 7;4(4):e0000802. doi: 10.1371/journal.pdig.0000802 (PMC11975109; doi:10.1371/journal.pdig.0000802)
Supplement: S1 Survey — (PDF) [file pdig.0000802.s003.pdf]

PPG 2022 Survey

Thank you for agreeing to participate in the @PandemicPregnancyGuide (PPG) Study. Please complete the following questions to the best of your ability. All questions are optional.

In this questionnaire, we define postpartum as the 1-year period following the birth of an infant.

Section 1.0 How do you find health information?

What have been your top 3 sources of pregnancy or postpartum-related health information during the pandemic (i.e. from March 2020 until present)? Select up to 3.

- ☐ Social circle (e.g. family, friends)
- ☐ Visiting your own family physician
- ☐ Another type of health professional that was not your own family physician (please specify)
- ☐ Print media (e.g. newspapers, magazines)
- ☐ Broadcast media (e.g. television, radio)
- ☐ Podcasts
- ☐ The Internet (e.g. Google, websites, blogs, etc.)
- ☐ Social media (e.g. Facebook, Twitter, Instagram)
- ☐ Email newsletters
- ☐ Other sources (please specify)
- ☐ Not applicable

Please specify the type of health professional

____

Please specify other sources

____

What have been your top 3 sources of COVID-19-related health information during the pandemic (i.e. from March 2020 until present)? Select up to 3.

- ☐ Social circle (e.g. family, friends)
- ☐ Visiting your own family physician
- ☐ Another type of health professional that was not your own family physician (please specify)
- ☐ Print media (e.g. newspapers, magazines)
- ☐ Broadcast media (e.g. television, radio)
- ☐ Podcasts
- ☐ The Internet (e.g. Google, websites, blogs, etc.)
- ☐ Social media (e.g. Facebook, Twitter, Instagram)
- ☐ Email newsletters
- ☐ Other sources (please specify)

Please specify the type of health professional

____

Please specify other sources

____

What were your top 3 sources of general health-related information (i.e. treating a sinus infection or pap smear information) before the pandemic (i.e. before March 2020)? Select up to 3.

- ☐ Social circle (e.g. family, friends)
- ☐ Visiting your own family physician
- ☐ Another type of health professional that was not your own family physician (please specify)
- ☐ Print media (e.g. newspapers, magazines)
- ☐ Broadcast media (e.g. television, radio)
- ☐ Podcasts
- ☐ The Internet (e.g. Google, websites, blogs, etc.)
- ☐ Social media (e.g. Facebook, Twitter, Instagram)
- ☐ Email newsletters
- ☐ Other sources (please specify)
- ☐ I cannot remember the sources I used

Please specify the type of health professional

____

Please specify other sources

____

Which social media platforms do you currently use for health information? This includes pregnancy, postpartum, and other health (including COVID-19) information. Select all that apply.

- ☐ Facebook
- ☐ YouTube
- ☐ Instagram
- ☐ Twitter
- ☐ Snapchat
- ☐ Pinterest
- ☐ LinkedIn
- ☐ TikTok
- ☐ Reddit
- ☐ Messaging apps (e.g. WhatsApp, WeChat)
- ☐ Other sources (please specify)
- ☐ Not applicable to me (i.e. I do not use social media platforms for health information)

Please specify other sources

____

We are interested in understanding your comfort with using digital tools for health information in general. For each statement, please indicate which response best reflects your experience right now.

(Norman, C.D., et al., eHEALS: The eHealth Literacy Scale. J Med Internet Res 2006;8(4):e27. Doi: 10.2196/jmir.8.4.e27)

	Strongly disagree	Disagree	Undecided	Agree	Strongly agree
I know what health resources are available on the Internet.	○	○	○	○	○
I know where to find helpful health resources on the Internet.	○	○	○	○	○
I know how to find helpful health resources on the Internet.	○	○	○	○	○
I know how to use the Internet to answer my questions about health.	○	○	○	○	○
I know how to use the health information I find on the Internet.	○	○	○	○	○
I have the skills I need to evaluate the health resources I find on the Internet.	○	○	○	○	○
I can tell high quality health resources from low quality health resources on the Internet.	○	○	○	○	○
I feel confident in using information from the Internet to make health decisions.	○	○	○	○	○

2.0 How and why have you used PPG?

How did you first hear about the @PandemicPregnancyGuide (PPG) Instagram account? Select all that apply.	☐ Friend/family member recommended the account<br>☐ A health care provider recommended the account<br>☐ Instagram recommended the account (e.g. I saw it on my feed)<br>☐ Can't remember how I first heard about PPG<br>☐ Other (please specify)
Please specify	__________________________________
In order to participate in this study, you indicated that you are a current or past follower of PPG.<br><br>Are you a current follower of PPG?	◯ Yes<br>◯ No
Approximately when did you start following PPG on Instagram?	◯ I approximately started following PPG...<br>◯ I can't remember when I first started following PPG
Year	◯ 2020<br>◯ 2021<br>◯ 2022<br>◯ 2023
Month	◯ January<br>◯ February<br>◯ March<br>◯ April<br>◯ May<br>◯ June<br>◯ July<br>◯ August<br>◯ September<br>◯ October<br>◯ November<br>◯ December

Approximately when did you remove yourself as a follower of PPG on Instagram?

Year	◯ 2020<br>◯ 2021<br>◯ 2022<br>◯ 2023
Month	◯ January<br>◯ February<br>◯ March<br>◯ April<br>◯ May<br>◯ June<br>◯ July<br>◯ August<br>◯ September<br>◯ October<br>◯ November<br>◯ December

Why did you remove yourself as a follower of PPG on Instagram? Select all that apply.	☐ I was no longer pregnant or postpartum (i.e. one year after giving birth).<br>☐ My children are no longer infants.<br>☐ PPG posted too frequently.<br>☐ PPG posted too infrequently.<br>☐ I stopped caring about COVID-19.<br>☐ Information about COVID-19 was too stressful for me.<br>☐ My friends/family unfollowed.<br>☐ I felt I had all the information I needed about pregnancy.<br>☐ I felt I had all the information I needed about COVID.<br>☐ I no longer found the information useful.<br>☐ I disagreed with the information posted.<br>☐ I found other social media accounts to follow.<br>☐ I did not have enough time to follow.<br>☐ I stopped following all healthcare related accounts.<br>☐ I stopped using social media.<br>☐ I can't remember why I removed myself as a follower.<br>☐ Other (please specify)
Please specify	________________________________

For the following set of questions, please refer to the attached screenshots shown below of some examples of the features used in PPG content.

To what extent did you find that our Instagram posts explaining scientific studies (such as the example below) helpful for learning new health information?

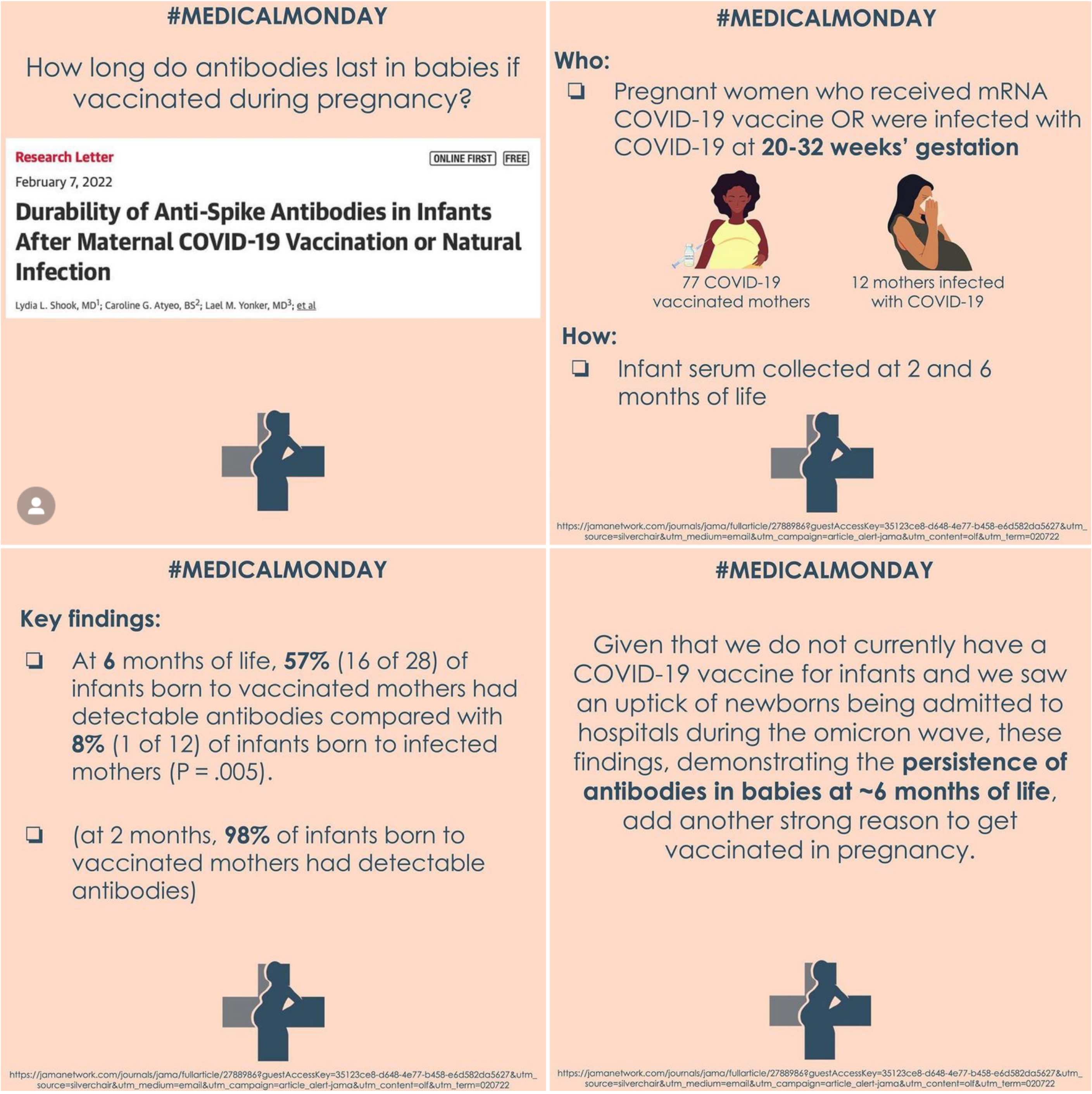

- ◯ Very helpful
- ◯ Helpful
- ◯ Neither helpful nor unhelpful
- ◯ Unhelpful
- ◯ Very unhelpful
- ◯ Not applicable or had not seen these posts

To what extent did you find the captions (text below the slides) of PPG posts helpful for learning new health information?

pandemicpregnancyguide During the COVID-19 pandemic, routine in school-based vaccination programs, such as the HPV (Human Papilloma Virus) vaccine have been temporarily suspended. The HPV vaccine has been shown to decrease the risk of cervical cancer. In honour of Canadian #cervicalcancerawarenessweek (October 25-30, 2021) and #MedicalMonday, we are sharing this letter by Dr. Milena Forte and our own PPG student @laurdiamond that addresses the importance of catch-up vaccination programs.

Teenagers who were scheduled to complete their vaccination schedule in 2020 or 2021 did not have access to their in school vaccinations due to the temporary suspension. Subsequently, attendance at catch-up vaccination clinics has been low. The main students affected by the suspension would have been in grade seven in the 2019/2020 and 2020/2021 academic years. "Adequate catch up vaccination is unlikely to happen without a coordinated, school-based approach."

It's also important to highlight that school-based vaccination programs allow for more equitable vaccine uptake. This is especially important given the well-established increased burden of cervical cancer and reduced cervical cancer screening in low-income communities.

We at @PandemicPregnancyGuide are advocating for women's health that the government and public health put a plan in place to catch up on the vaccination schedule.

pandemicpregnancyguide Bottom line: Cervical cancer is a vaccine preventable disease. The HPV vaccine is safe and effective at preventing cervical cancer for all people, regardless of age, who are sexually active or plan on becoming sexually active. School-based vaccination programs must be reinstated and prioritized. Specialized timing in relation to the COVID vaccine does not need to be coordinated.

For more information on HPV, check out this resource from the Society of Obstetricians and Gynaecologists of Canada: https://www.hpvinfo.ca/

Full study text here:
https://www.cmaj.ca/content/cmaj/193/37/E1467.full.pdf

Disclaimer: The general information provided on this account is for informational purposes only and is not professional medical advice, diagnosis, treatment, or care, nor is it intended to be a substitute therefore. Always seek the advice of your healthcare provider in your jurisdiction to discuss any questions that you believe may be relevant to you or to someone else.

◯ Very helpful
◯ Helpful
◯ Neither helpful nor unhelpful
◯ Unhelpful
◯ Very unhelpful
◯ Not applicable or had not seen these posts

To what extent did you find Instagram Live sessions (for example Q&As with medical experts or guided activities, with the ability to ask real-time questions), helpful for learning new health information?

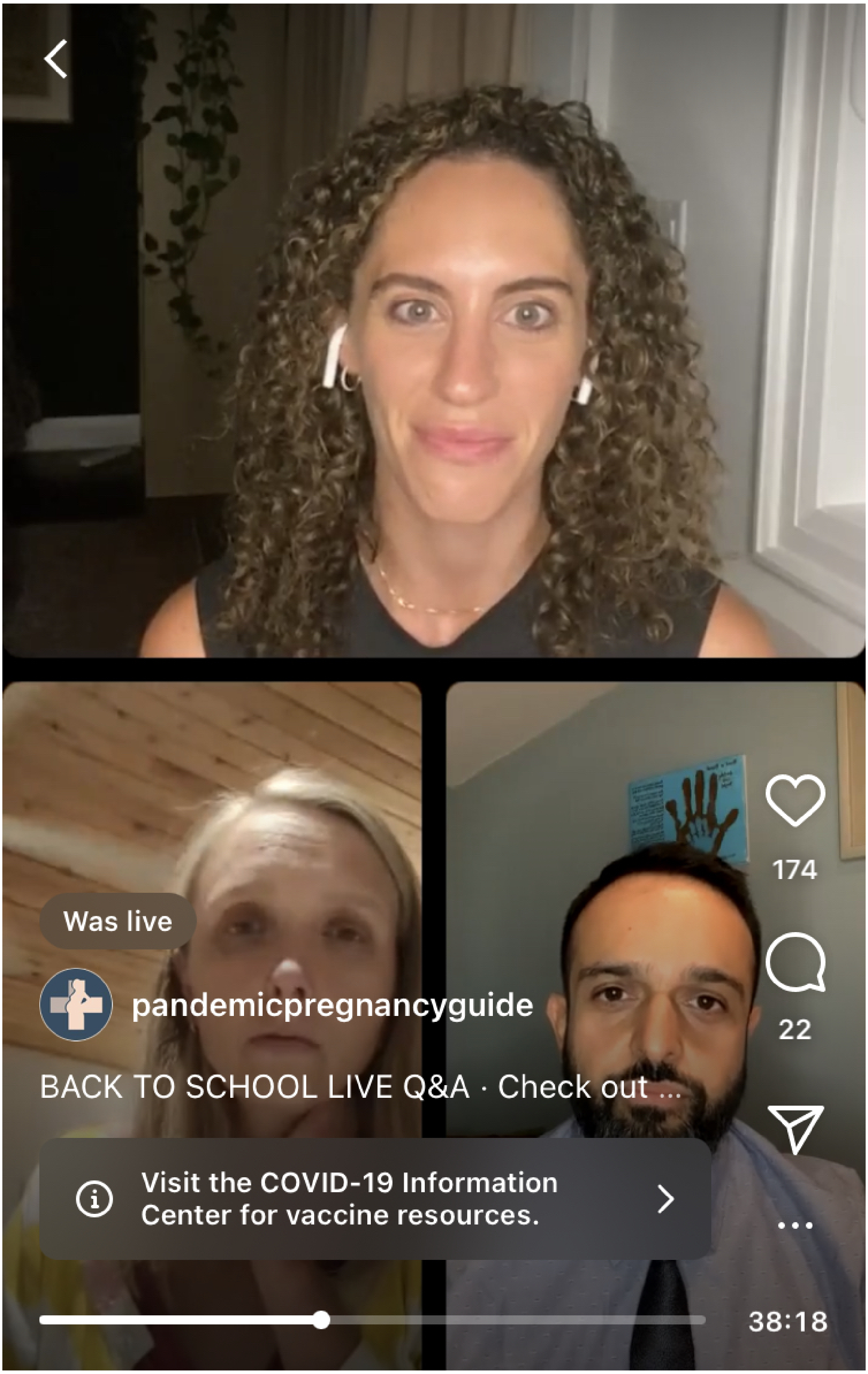

◯ Very helpful
◯ Helpful
◯ Neither helpful nor unhelpful
◯ Unhelpful
◯ Very unhelpful
◯ Not applicable or had not seen these posts

To what extent did you find recorded videos (e.g. interviews with medical experts) helpful for learning new health information?

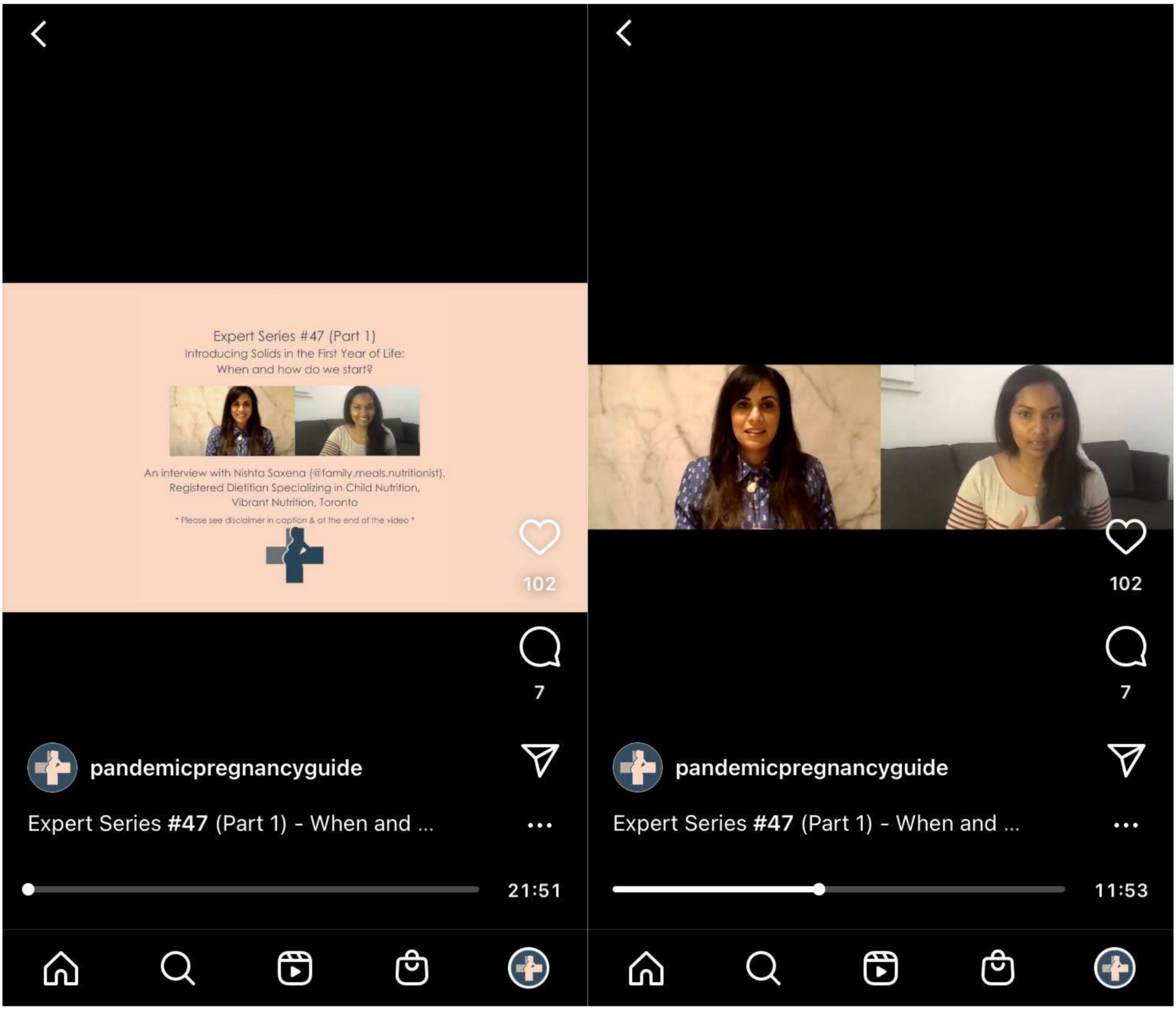

◯ Very helpful
◯ Helpful
◯ Neither helpful nor unhelpful
◯ Unhelpful
◯ Very unhelpful
◯ Not applicable or had not seen these posts

To what extent do you find Instagram stories (e.g. answering FAQs or announcing a new post) helpful for finding or learning new health information?

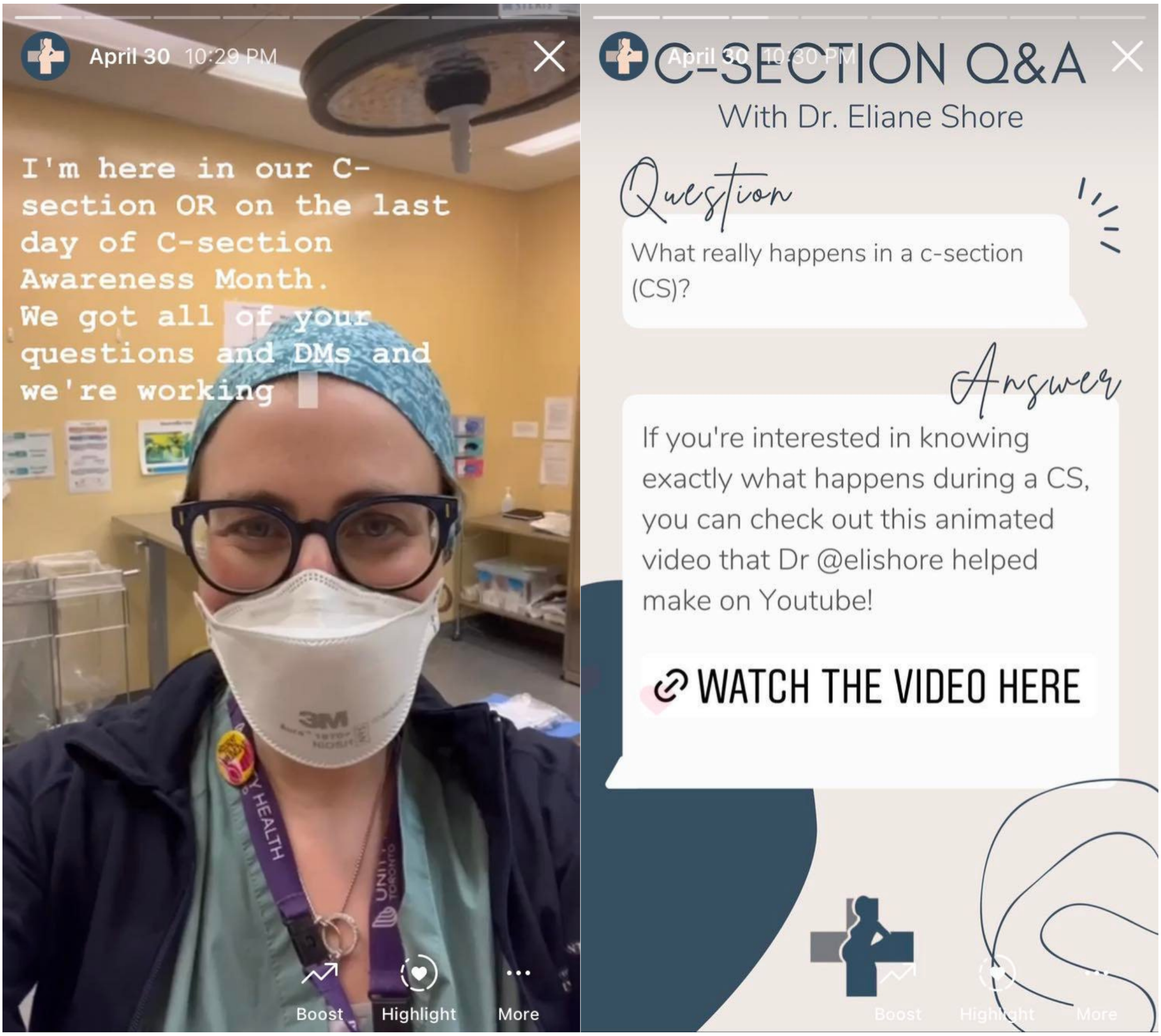

◯ Very helpful
◯ Helpful
◯ Neither helpful nor unhelpful
◯ Unhelpful
◯ Very unhelpful
◯ Not applicable or had not seen these posts

How do you prefer to access PPG? Select all that apply.	☐ I prefer viewing / reading Instagram posts with slides<br>☐ I prefer viewing Instagram videos/reels<br>☐ I prefer viewing / reading Instagram stories<br>☐ I am unsure of the difference between posts, video/reels, and stories on Instagram<br>☐ I would prefer to access future content not on social media (e.g. monthly email, website)<br>☐ I prefer something else (please specify)
Please specify	______________________________

We would like to understand your experience reading PPG posts. A PPG post is below as an example. Please look at the example post and describe your agreement with the following statements in regards to PPG medical posts in general. If you are completing this survey on a computer, you can right click the image below to zoom into the text.

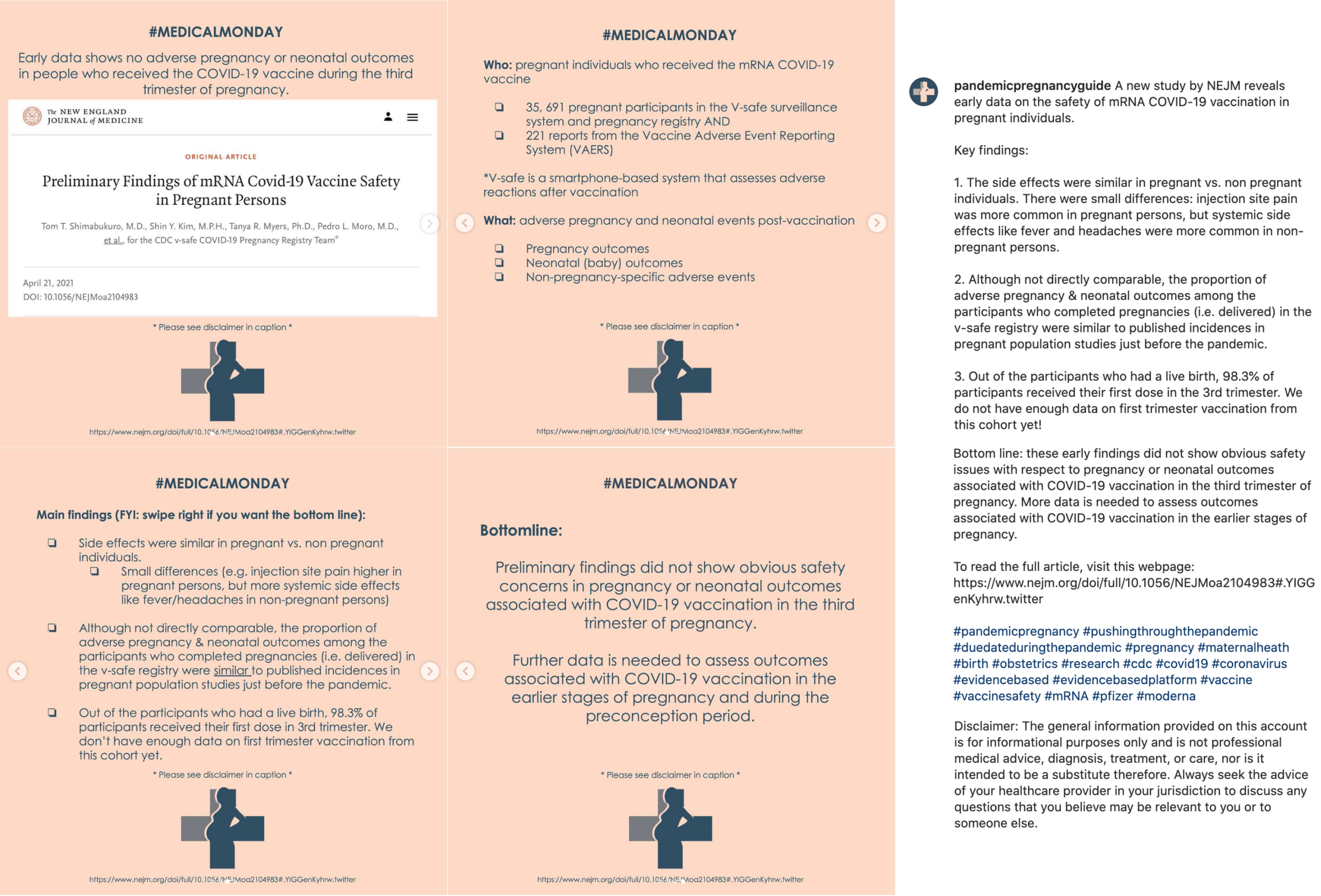

	Strongly disagree	Disagree	Undecided	Agree	Strongly agree
I understood the science content on PPG.	○	○	○	○	○
There is too much science language on PPG's posts.	○	○	○	○	○

I appreciated knowing the methods of a study shared in PPG's posts (i.e. how the authors reached their conclusion).	◯	◯	◯	◯	◯
I skip to the end of a post to read the takeaway points.	◯	◯	◯	◯	◯
I found the health information provided by PPG helpful.	◯	◯	◯	◯	◯

Why did you find (or not find) PPG posts to be helpful? (Optional)

____

We are interested in knowing your thoughts on the accessibility of our @PandemicPregnancyGuide (PPG) content. Please indicate whether the following statements apply to you.

PPG has sufficient subtitles for audio content.
◯ Yes
◯ No

PPG uses a font size that is large enough for me to read.
◯ Yes
◯ No

I use a screen reader when accessing social media.
◯ Yes
◯ No

I have difficulty making out the images (i.e. the color contrast of posts is poor).
◯ Yes
◯ No

I am able to swipe through the different images on a post if I choose to do so.
◯ Yes
◯ No

I am able to find the original source of information if I choose to do so.
◯ Yes
◯ No

I am able to understand most of the words used in the posts.
◯ Yes
◯ No

Please describe your level of agreement with the following statements.

	Strongly disagree	Disagree	Undecided	Agree	Strongly agree
PPG was a reliable source of health information.	○	○	○	○	○
PPG made it easier for me to understand health information.	○	○	○	○	○
PPG allowed me to be up to date on public health policy decisions related to COVID-19.	○	○	○	○	○
PPG allowed me to be up to date on new research related to pregnancy or postpartum during COVID-19.	○	○	○	○	○
PPG provided me with health information that I was not able to obtain from my healthcare provider.	○	○	○	○	○
The information shared on PPG is truthful.	○	○	○	○	○
I trust PPG not to share or spread misinformation.	○	○	○	○	○
Reading PPG helped dispel misinformation from other sources (including online, friends or family, etc.).	○	○	○	○	○
PPG did not help me navigate my health experiences during the pandemic .	○	○	○	○	○
I valued being a part of the PPG community.	○	○	○	○	○
By following PPG, I felt like I was not alone in my experiences during the pandemic.	○	○	○	○	○
Information provided by PPG allowed me to be less worried/anxious during my pregnancy journey.	○	○	○	○	○
I often shared interesting posts from PPG on my story or with friends.	○	○	○	○	○

On a scale from 0 (not likely at all) to 100 (extremely likely), how likely would you be to recommend PPG to a friend or colleague? 50 represents being neither likely nor unlikely.

Not likely at all — Neither likely nor unlikely — Extremely likely

(Place a mark on the scale above)

Did it matter to you that PPG was run by physicians?	◯ Yes<br>◯ No
Did it matter to you that PPG was run by Canadian physicians with Canadian specific content?	◯ Yes<br>◯ No
Did you also follow PPG for its online community of support during the pandemic?	◯ Yes<br>◯ No

3.0 What health topics are of interest or concern to you?

Think back to the time when you relied the most heavily on PPG for health information. Approximately when was this time period?

- ◯ April-June 2020
- ◯ July-September 2020
- ◯ October-December 2020
- ◯ January-March 2021
- ◯ April-June 2021
- ◯ July-September 2021
- ◯ October-December 2021
- ◯ January-March 2022
- ◯ April-June 2022
- ◯ July-September 2022
- ◯ October 2022-present
- ◯ Other (please specify)
- ◯ I do not remember the time period

Please specify ______________________________

Thinking back to the time when you relied the most heavily on PPG for health information, which topics were you most interested in reading about? Select all that apply.

- ☐ COVID-19 preventative measures (e.g. masking, social distancing)
- ☐ COVID-19 vaccination while trying to conceive
- ☐ COVID-19 vaccination while pregnant
- ☐ COVID-19 vaccination while breastfeeding
- ☐ COVID-19 vaccination in children
- ☐ COVID-19 infection while try to conceive
- ☐ COVID-19 infection while pregnant
- ☐ COVID-19 infection while breastfeeding
- ☐ COVID-19 infection in newborns and children
- ☐ Impact of COVID-19 on child development (e.g. school closures)
- ☐ COVID-19 variants
- ☐ Labour and delivery during COVID-19
- ☐ General women's and/or sexual health (non-COVID-19 related)
- ☐ Pregnancy and postpartum topics (non-COVID-19 related)
- ☐ Labour and delivery (non-COVID-19 related)
- ☐ Infant and child health (non-COVID-19 related)
- ☐ Personal wellness (e.g. fitness, nutrition, sleep)
- ☐ Mental health
- ☐ Other topics (please specify)

Please specify ______________________________

Thinking back to the time when you relied the most heavily on PPG for health information, were there health topics that we did not cover on PPG during that time that you wanted more information on?

- ◯ Yes (please specify)
- ◯ No

Please specify ______________________________

Please indicate your level of agreement with the following statements regarding your decisions during the COVID-19 pandemic (i.e. from March 2020 until present).

Because of the information I received from PPG, I was more likely to ...

	Strongly disagree	Disagree	Undecided	Agree	Strongly agree	Not applicable to me
Use COVID-19 preventative measures (e.g. masking, social distancing) while pregnant.	○	○	○	○	○	○
Practice COVID-19 preventative measures around those who were not yet eligible for the vaccine or more vulnerable (e.g. newborns, elderly, immunocompromised).	○	○	○	○	○	○
Get the first 2 doses of the COVID-19 vaccination for myself.	○	○	○	○	○	○
Get the COVID-19 vaccine booster(s) for myself.	○	○	○	○	○	○
Get the COVID-19 vaccine during pregnancy.	○	○	○	○	○	○
Get the COVID-19 vaccine while trying to conceive or breastfeeding.	○	○	○	○	○	○
Encourage my social circle (e.g., partner, family, friends) to get the COVID-19 vaccine.	○	○	○	○	○	○
Ensure my child(ren) got (or will get) the COVID-19 vaccine when approved and available for them.	○	○	○	○	○	○
Feel comfortable continuing to breastfeed while having COVID-19 infection.	○	○	○	○	○	○

Think about the next year in your life. Which topics would you like to see covered on PPG? Select all that apply.

- ☐ General women's and/or sexual health
- ☐ Pregnancy and postpartum topics
- ☐ Labour and delivery
- ☐ Infant and child health
- ☐ Parenting support and guidance
- ☐ Personal wellness (e.g. fitness, nutrition, sleep)
- ☐ Navigating interpersonal relationships (e.g. marital, friendships, familial, etc.)
- ☐ Mental health
- ☐ Emerging viruses
- ☐ Impact of the pandemic on child development (e.g. academic and social catch up)
- ☐ Impact of global supply chain issues on maternal and child health (e.g. formula, epidural, acetaminophen/Tylenol shortage)
- ☐ Impact of the economy on maternal and child health (e.g. inflation)
- ☐ Safety/risks of various medications, substances, treatments, and activities while pregnant or breastfeeding (e.g. acetaminophen, marijuana, acupuncture, and hair coloring, etc.)
- ☐ COVID-19 preventative measures (e.g. masking, social distancing)
- ☐ COVID-19 vaccination while trying to conceive, pregnant, postpartum, or breastfeeding
- ☐ COVID-19 infection while trying to conceive, pregnant, postpartum, or breastfeeding
- ☐ COVID-19 vaccination in children
- ☐ COVID-19 infection in newborns and children
- ☐ COVID-19 variants
- ☐ Other topics (please specify)

Please specify

____

4.0 Questions about you

The following questions will help us to understand who uses PPG. This is important as it will allow us to contextualize our results and also identify groups of people who received, and did not receive information from the PPG account.

What year were you born?

____

What gender do you most closely identify with?

- ◯ Woman
- ◯ Intersex
- ◯ Non-Binary (genderqueer)
- ◯ Transgender
- ◯ Gender Fluid
- ◯ Questioning
- ◯ Two-spirit
- ◯ Man
- ◯ Do not know
- ◯ I prefer to self-describe
- ◯ Prefer not to answer

Please describe

____

What is your sexual orientation?

- ◯ Heterosexual/straight
- ◯ Gay, Lesbian, or Queer
- ◯ Bi-sexual
- ◯ Pansexual
- ◯ Asexual
- ◯ Do not know
- ◯ I prefer to self-describe
- ◯ Prefer not to answer

Please describe

____

Which of the following best describes your race or ethnic group? Please check ALL that apply.	☐ Indigenous (e.g., First Nations, Inuit, Métis person from any country)<br>☐ White - European (British, German, Swedish & others)<br>☐ White - North American (eg., American, Canadian, Mexican & others)<br>☐ Black - North American (eg., American, Canadian, Mexican &others)<br>☐ Black - South and Central American (e.g., Panamanian, Nicaraguan & others)<br>☐ Black - Afro-Caribbean (e.g., Haitian, Dominican, Jamaican & others)<br>☐ Black - Afro-European (e.g., British, German, Spanish & others)<br>☐ Black - African (e.g., Ghana, Nigeria, Kenyan, Tanzanian & others)<br>☐ LatinX or Hispanic - Central American (e.g., Costa Rican, Nicaraguan & others)<br>☐ LatinX or Hispanic - South American (e.g., Brazilian, Chilean, Colombian & others)<br>☐ LatinX or Hispanic - Caribbean (e.g., Jamaican, Dominican, Bahamian & others)<br>☐ LatinX or Hispanic - European (e.g., Spanish, Portuguese & others)<br>☐ Middle Eastern - North African (e.g., Algerian, Egyptian, Libyan, Moroccan & others)<br>☐ Middle Eastern - Middle Eastern/West Asian (e.g., Anatolian, Arabian, Levantines & others)<br>☐ Southeast Asian - Filipino<br>☐ Southeast Asian - Other (e.g., Cambodian, Indonesian, Laotian, Vietnamese, & others)<br>☐ South Asian - East Indian, Pakistani, Sri Lankan, & others<br>☐ East Asian - Chinese<br>☐ East Asian - Japanese<br>☐ East Asian - Korean<br>☐ East Asian - Mongolian, Taiwanese, & others<br>☐ West Asian - Afghan, Iranian, Turkish, & others<br>☐ Central Asian - Kazakh, Uzbek, & others<br>☐ I prefer to self-describe<br>☐ Prefer not to answer
Please describe	______________________________
Do you identify as a 'racialized person' (defined as persons in Canada, other than Indigenous peoples, who are non-Caucasian in race or non-white in colour, regardless of place of birth or citizenship)?	◯ Yes<br>◯ No<br>◯ Not sure<br>◯ Prefer not to answer
What country do you currently live in?	◯ Canada<br>◯ United States<br>◯ Other (please specify)

What province do you live in?	◯ Newfoundland and Labrador<br>◯ Nova Scotia<br>◯ Prince Edward Island<br>◯ New Brunswick<br>◯ Quebec<br>◯ Ontario<br>◯ Manitoba<br>◯ Saskatchewan<br>◯ Alberta<br>◯ British Columbia<br>◯ Yukon<br>◯ Northwest Territories<br>◯ Nunavut
Please specify	__________________________________
How would you describe the area you live in?	◯ Rural<br>◯ Semi-urban<br>◯ Urban
What is the highest level of education you have completed?	◯ High school or less (primary or secondary education)<br>◯ Post-secondary education or training (college diploma, trade school or apprenticeship)<br>◯ Bachelor's university degree (or equivalent level)<br>◯ Graduate or professional university degree (or equivalent level)<br>◯ Prefer not to answer
What was your total annual household income (before taxes) last year (in CAD)?	◯ Less than $30,000<br>◯ $30,000 to $59,999<br>◯ $60,000 to $89,999<br>◯ $90,000 to $119,999<br>◯ $120,000 to $149,999<br>◯ More than $150,000<br>◯ Do not know<br>◯ Prefer not to answer
How many people does your household income support (including yourself)?	__________________________________
What is your current employment status?	◯ Working full-time<br>◯ Working part-time (< 30 hours/week)<br>◯ Currently on leave from work<br>◯ Stay-at-home parent<br>◯ Unemployed<br>◯ Student<br>◯ Other (please specify)<br>◯ Prefer not to answer
Please specify	__________________________________
Are you currently on...	◯ Parental leave<br>◯ Medical or disability leave<br>◯ Other (please specify)<br>◯ Prefer not to answer

Please specify

____

How would you rate your overall health?

- ◯ Very Good
- ◯ Good
- ◯ Fair
- ◯ Poor
- ◯ Very Poor

Have you been vaccinated against COVID-19? Answer 'Yes' if you received at least one dose of the COVID-19 vaccine.

- ◯ Yes
- ◯ No

How many doses of the COVID-19 vaccine have you received so far?

- ◯ 1
- ◯ 2
- ◯ 3
- ◯ 4
- ◯ 5+

At the time of your 1st or 2nd COVID-19 vaccine, were you:

- ◯ Thinking of trying to get pregnant in the next 6-12 months
- ◯ Trying to get pregnant
- ◯ Pregnant
- ◯ Breastfeeding
- ◯ None of the above

At the time of your 3rd or 4th vaccine COVID-19, were you:

- ◯ Thinking of trying to get pregnant in the next 6-12 months
- ◯ Trying to get pregnant
- ◯ Pregnant
- ◯ Breastfeeding
- ◯ None of the above
- ◯ Not applicable (i.e. I did not receive more than 2 COVID-19 vaccines)

Have you received a bivalent booster? (i.e. a booster dose of the COVID vaccine that targets 2 strains of the COVID virus)

- ◯ Yes
- ◯ No

How many times have you...

Been pregnant (please include all pregnancies, including those that ended prior to a live birth):

____

Had a live birth:

____

Did you conceive, deliver or adopt a child during the pandemic (March 2020 to present)?

- ◯ Yes
- ◯ No

What best describes your (and/or your partner's) current reproductive status at the time of completing this survey?

- ◯ Trying to conceive a child
- ◯ Currently pregnant
- ◯ In the postpartum period
- ◯ None of the above

What is your estimated date of delivery?

Month	◯ January<br>◯ February<br>◯ March<br>◯ April<br>◯ May<br>◯ June<br>◯ July<br>◯ August<br>◯ September<br>◯ October<br>◯ November<br>◯ December
Year	◯ 2024<br>◯ 2023
When was your youngest living child born?	
Month	◯ January<br>◯ February<br>◯ March<br>◯ April<br>◯ May<br>◯ June<br>◯ July<br>◯ August<br>◯ September<br>◯ October<br>◯ November<br>◯ December
Year	◯ 2023<br>◯ 2022<br>◯ 2021<br>◯ 2020
How many children currently live with you at least 50% of the time?	______________________________
What are the ages of your children? Please indicate how many of your children fall into each of the age ranges below.	
Infant (< 12 months)	______________________________
Toddler (1-3 years old)	______________________________
Preschool or Kindergarten (4-6 years old)	______________________________
Elementary school or older (>6 years old)	______________________________

5.0 Other

What do you like most about PPG?

____

What do you like the least about PPG? What could be improved?

____

Please describe anything else that you would like us to know about your use of the @PandemicPregnancyGuide Instagram account. You don't have to write anything if you don't want to.

____
