# Supplementary material for: Evaluation of a Canadian social media platform for communicating perinatal health information during a pandemic
Source: PLOS Digit Health. 2025 Apr 7;4(4):e0000802. doi: 10.1371/journal.pdig.0000802 (PMC11975109; doi:10.1371/journal.pdig.0000802)
Supplement: S1 Data — (DOCX) [file pdig.0000802.s004.docx]

**Appendix 3 – Additional Study Data**

**Appendix Table 3.1** Participants electronic health literacy score measured with the eHEALS (N=1818)

| **eHEALS Question** | **Mean** | **Standard Deviation** |
| --- | --- | --- |
| I know what health resources are available on the Internet | 3.193 | 0.706 |
| I know where to find helpful health resources on the Internet | 3.208 | 0.715 |
| I know how to find helpful health resources on the Internet | 3.241 | 0.698 |
| I know how to use the Internet to answer my health questions | 3.257 | 0.699 |
| I know how to use the health information I find on the Internet to help me | 3.189 | 0.713 |
| I have the skills I need to evaluate the health resources I find on the Internet | 3.235 | 0.785 |
| I can tell high quality from low quality health resources on the Internet | 3.291 | 0.736 |
| I feel confident in using information from the Internet to make health decisions | 2.859 | 0.887 |
| **Total Score** | **28.60** | **5.43** |

**Note:** E-health literacy score is calculated from the sum of 8 questions, which are each measured on a 5-point scale in the range from 1 (strongly disagree) to 5 (strongly agree). The overall range of the eHEALS score varies from 8 to 40, with a higher score indicating more perceived skills in finding, evaluating, and using electronic information to make health decisions.

**Appendix Table 3.2** Engagement with PPG across the pandemic (March 2020 to April 2023).

|  | **n (%)** | |
| --- | --- | --- |
| **Current follower** (n=1815) | 1734 | (96) |
| **Timing of follow** (n=1462) |  |  |
| Prior to July 2020 | 586 | (40.1) |
| July-October 2020 | 230 | (15.7) |
| November 2020-February 2021 | 268 | (18.3) |
| March-June 2021 | 190 | (13.0) |
| July-October 2021 | 87 | (6.0) |
| November 2021-February 2022 | 66 | (4.5) |
| March-June 2022 | 18 | (1.2) |
| July-October 2022 | 14 | (1.0) |
| November 2022-April 2023 | 3 | (0.2) |
| **Timing of unfollow** (n=66) |  |  |
| March-June 2020 | 0 | (0.0) |
| July-October 2020 | 9 | (13.6) |
| November 2020-February 2021 | 7 | (10.6) |
| March-June 2021 | 8 | (12.1) |
| July-October 2021 | 8 | (12.1) |
| November 2021-February 2022 | 11 | (16.7) |
| March-June 2022 | 5 | (7.6) |
| July-October 2022 | 6 | (9.1) |
| November 2022-April 2023 | 12 | (18.2) |
| **Timing of most use** (n=1818) |  |  |
| April-June 2020 | 361 | (19.9) |
| July-September 2020 | 221 | (12.2) |
| October-December 2020 | 164 | (9.0) |
| January-March 2021 | 259 | (14.2) |
| April-June 2021 | 261 | (14.4) |
| July-September 2021 | 103 | (5.7) |
| October-December 2021 | 128 | (7.0) |
| January-March 2022 | 103 | (5.7) |
| April-June 2022 | 29 | (1.6) |
| July-September 2022 | 18 | (1.0) |
| October 2022-April 2023 | 22 | (1.2) |
| Other, or don’t remember | 149 | (8.2) |

**Appendix Table 3**.**3** Participant reported experience with PPG

|  | **Agree or**  **Strongly Agree** | | **Undecided** | | **Disagree or**  **Strongly Disagree** | |
| --- | --- | --- | --- | --- | --- | --- |
| **Experience with PPG Posts, n (%)** | | | | | | |
| I understood the science content (n=1811) | 1764 | (97.4) | 34 | (1.9) | 13 | (0.7) |
| There is too much science language (n=1814) | 88 | (4.9) | 130 | (7.2) | 1596 | (87.9) |
| I appreciate knowing the methods of a study (n=1807) | 1709 | (94.5) | 80 | (4.4) | 18 | (1.0) |
| I skip to the end to read the takeaway points (n=1815) | 380 | (21.0) | 288 | (15.9) | 1147 | (63.2) |
| I found the health information provided by PPG helpful (n=1811) | 1798 | (99.3) | 10 | (0.6) | 3 | (0.2) |
| **General experience with PPG, n (%)** | | | | | | |
| [PPG is] a reliable source of information (n=1801) | 1786 | (99.2) | 0 | (0.0) | 1 – 5 | (< 0.3) |
| [PPG] made it easier to understand health information (n=1798) | 1769 | (98.4) | 0 | (0.0) | 1 – 5 | (< 0.3) |
| [PPG] allowed me to be up to date on public health policy related to COVID-19 (n=1806) | 1764 | (97.7) | 3 | (0.2) | 1 – 5 | (< 0.3) |
| Allowed me to be up to date on research related to Pregnancy/postpartum during COVID-19 (n=1806) | 1788 | (99.1) | 15 | (0.8) | 1 – 5 | (< 0.3) |
| [PPG] provide[d] health information I could not obtain from my healthcare provider (n=1808) | 1246 | (68.9) | 372 | (20.6) | 190 | (10.5) |
| The information shared [on PPG] is truthful (n=1808) | 1786 | (99.2) | 20 | (1.1) | 1 – 5 | (< 0.3) |
| I trust PPG not to share/spread misinformation (n=1801) | 1779 | (99.0) | 20 | (1.1) | 1 – 5 | (< 0.3) |
| Reading PPG helped dispel misinformation from other sources (n=1798) | 1660 | (92.4) | 115 | (6.4) | 23 | (1.3) |
| PPG did not help me navigate my health experiences during the pandemic (n=1811) | 143 | (7.9) | 59 | (3.3) | 1609 | (88.9) |
| I value being in the PPG community (n=1810) | 1564 | (86.5) | 235 | (13.0) | 11 | (0.7) |
| By following PPG I felt like I was not alone in my experiences during the pandemic (n=1807) | 1636 | (90.6) | 149 | (8.2) | 22 | (1.2) |
| Information from PPG allowed me to be less worried/anxious during my pregnancy journey (n=1804) | 1578 | (87.5) | 186 | (10.3) | 40 | (2.2) |
| I often shared interesting posts on my story or with friends (n=1806) | 1169 | (64.7) | 253 | (14.0) | 384 | (21.3) |

**Appendix Table 3.4** Reading Level of Medical Monday Posts

| **Date** | **Topic** | **Flesch Reading Ease Score** | **Flesch-Kincaid Grade Level** |
| --- | --- | --- | --- |
| Oct 5 2020 | Delaying pregnancy during COVID-19 | 12.7 | 21 |
| Oct 19 2020 | COVID-19 transmission from mother to neonate | 0 | 27.3 |
| Nov 9 2020 | Epidural and autism risk | 32.2 | 13.3 |
| Dec 7 2020 | COVID-19 pregnancy and neonate outcomes | 22.2 | 18.7 |
| Dec 14 2020 | COVID-19 vaccine and pregnancy/breastfeeding | 7.4 | 26.6 |
| Dec 21 2020 | COVID-19 vaccine and pregnancy/breastfeeding | 11.2 | 24.6 |
| Jan 11 2021 | COVID-19 vaccine and conception | 17 | 19.3 |
| Jan 18 2021 | COVID-19 infection impact on delivery | 0 | 26.7 |
| Feb 1 2021 | COVID-19 antibodies | 16.5 | 23.5 |
| Feb 8 2021 | COVID-19 antibodies | 9 | 27.1 |
| Feb 15 2021 | COVID-19 severity in pediatric pop. | 19.5 | 20.6 |
| March 1 2021 | COVID-19 vaccine and fertility | 11.7 | 21.1 |
| March 15 2021 | Moderna vaccine safety children | 28.6 | 16.4 |
| March 22 2021 | COVID-19 pregnancy and neonate outcomes | 22.4 | 20 |
| March 22 2021 | COVID-19 antibodies in pregnancy | 0.6 | 25.7 |
| March 29 2021 | COVID-19 antibodies | 0 | 32.7 |
| April 12 2021 | COVID-19 pregnancy and neonate outcomes | 7.7 | 22.6 |
| April 26 2021 | COVID-19 vaccine | 32.9 | 14 |
| May 3 2021 | COVID-19 vaccine | 25.3 | 13.9 |
| May 17 2021 | IUD use as emergency contraception | 18.7 | 21.2 |
| May 31 2021 | COVID-19 antibodies | 24.5 | 18.7 |
| June 7 2021 | COVID-19 vaccine | 27.9 | 16.7 |
| June 21 2021 | Male fertility | 25.4 | 16.9 |
| June 28 2021 | Long distance travel for birth | 0 | 26.5 |
| July 5 2021 | Virtual care during pandemic | 0 | 32 |
| July 19 2021 | COVID-19 vaccine | 4.4 | 21.5 |
| July 27 2021 | Sexual health during COVID-19 | 19.9 | 19.3 |
| Aug 2 2021 | Induction of labour | 23 | 18.4 |
| Aug 16 2021 | COVID-19 vaccine | 20.7 | 18.8 |
| Aug 23 2021 | COVID-19 vaccine | 0 | 33.9 |
| Sept 13 2021 | COVID-19 infection during pregnancy | 7.3 | 19.1 |
| Sept 20 2021 | Pregnancy during COVID-19 | 34.4 | 15.8 |
| Sept 27 2021 | Tylenol use in pregnancy | 0 | 35 |
| Oct 11 2021 | Cannabis use in pregnancy | 19.1 | 18.2 |
| Oct 18 2021 | COVID-19 vaccine | 14.5 | 18.5 |
| Oct 25 2021 | HPV Vaccine | 22.5 | 15 |
| Nov 22 2021 | COVID-19 vaccine | 26.6 | 16.3 |
| Nov 29 2021 | Omicron | 36.9 | 15.5 |
| Dec 6 2021 | COVID-19 antibodies | 24.5 | 15.6 |
| Jan 3 2022 | COVID-19 vaccine | 0 | 35.1 |
| Jan 10 2022 | COVID-19 vaccination and menstrual cycles | 19.3 | 19.5 |
| Jan 24 2022 | COVID-19 vaccine | 6.1 | 20.1 |
| Jan 31 2022 | Breastfeeding and COVID-19 infection | 53.7 | 10.5 |
| Feb 7 2022 | COVID-19 studies | 27.4 | 16 |
| Feb 14 2022 | Infant COVID-19 antibodies with maternal vaccination | 0 | 30.5 |
| June 13 2022 | COVID-19 vaccination during pregnancy | 8.5 | 19.7 |
| Aug 1 2022 | Neurodevelopment outcomes and COVID-19 infection | 32.2 | 14.9 |
| Oct 3 2022 | COVID-19 antibodies | 15.3 | 24.4 |
| Oct 24 2022 | COVID-19 vaccination and menstrual cycles | 32.6 | 13.7 |
| Nov 7 2022 | Antidepressant use in pregnancy | 1.8 | 21.9 |
| Jan 18 2023 | Domperidone and breastfeeding | 17.4 | 18.8 |
| March 5 2023 | Alcohol Use guidelines | 26.9 | 15.8 |

**Appendix Table 3.5** Engagement with PPG Posts

|  | **Likes per post**  (n = 442) | **Comments per post**  (n=442) | **Shares per post**  (n=323)^1^ | **Saves per post**  (n=442) | **Views per post**  (n = 126)^2^ |
| --- | --- | --- | --- | --- | --- |
| **Median** | **190** | **9** | **2** | **30** | **3374** |
| [Q1-Q3] | [87 –415] | [3 – 25] | [0 – 38] | [12 – 78] | [2542 – 5376] |
| **Mean**  (SD) | **348**  (436) | **23**  (72) | **93**  (331) | **84**  (265) | **5075**  (5905) |
| **Range** | **3213** | **1395** | **4641** | **4481** | **54323** |
| Minimum | 19 | 0 | 0 | 0 | 192 |
| Maximum | 3232 | 1395 | 4641 | 4481 | 54515 |

1 Data collection on this feature was not available for the earlier content; data reported reflects content from August 2020 to November 2023

2 Views only applies to video content
