# Supplementary material for: Evaluation of a Canadian social media platform for communicating perinatal health information during a pandemic
Source: PLOS Digit Health. 2025 Apr 7;4(4):e0000802. doi: 10.1371/journal.pdig.0000802 (PMC11975109; doi:10.1371/journal.pdig.0000802)
Supplement: S1 Codebook — (DOCX) [file pdig.0000802.s005.docx]

**Appendix 3 – Qualitative Codebook**

**Appendix Table 3.1**. Codebook, Results of Qualitative Content Analysis, Predominant Themes

| **Participants Values Aspects of @PandemicPregnancyGuide** | | | |
| --- | --- | --- | --- |
| **Theme** | **N = 1267**  **n (%)** | **Description** | **Example Quotes** |
| **Trustworthy or Reliable** | 454 (36) | PPG was a resource they could trust or rely on to provide accurate information about pregnancy and COVID. | *“I am an [healthcare provider] who was new to practice during the pandemic- I have friends and family as well as patient[s] who were pregnant, TTC, or post-partum. The posts with information and links to reliable scholarly articles were utilized many times in my practice. These articles were then shared with people so they might get information from an appropriate source. It is hard to find trustworthy information on the internet and I very much appreciate having somewhere to direct patients, family, and friends.” – P1631* |
|  |  |  | *“Canadian content from a trusted Toronto hospital. I gave birth June 2020. And so much of the practical information required during Covid (can I bring my partner to the hospital, Will there be food in the hospital) was US based and unhelpful for parents in Canada with a different political landscape, healthcare and resources re: Covid. I appreciated the empirical research as well, given all the information floating around so early in the pandemic, when parental anxiety was at its peak. I trusted Talia with the info I was receiving, and it was so relevant to my personal journey at that time. Canadian content, that is empirically based - this info became my guide and gold standard during the pandemic, I saw studies on Instagram before my own doctor knew about them!” – P1950* |
|  |  |  | *“I like that I can trust the information being presented. It truly helped me feel less worried/ anxious and more informed while pregnant when the pandemic started. I found it extremely helpful after giving birth as well for general information about the vaccine and also specific info about the vaccine while breastfeeding. I appreciated you passing along recent studies and information about COVID-19 in a timely matter. I also love how understanding and compassionate you were toward pregnant/post-partum people during the early phases and waves of the pandemic. Thank you!!” – P467* |
| **Evidence Based** | 440 (35) | Valuing that PPG provided information based on research and evidence. | *“I liked that the posts were all cited, and research based as well. PPG also helped me decided to get my baby vaccinated when she was old enough, again thanks to the clear and research backed information” – P449* |
|  |  |  | *“Credible information about the impact of COVID-19 on pregnant people, breast-feeding, people, and newborns. I very much appreciate the links to the primary literature, and the dissection of the papers discussed.” – P2326* |
|  |  |  | *“I loved that you describe how the data was collected, what the results truly show/the accuracy, and summarize it. You give links and sources, and it just feels so credible. It would honestly make me feel so much better and when I was really anxious, I'd go to your page, and read something and be like "okay, this is the information I need to make a choice/feel better" and I'd stop there and try not to continue to cycle. I shared it with friends and family and felt confident doing so. I also enjoyed reading the comments and support from other moms, or the replies to others who were spreading fake news/arguing because it provided me responses to things I was reading/hearing that would make me question things.” – P281* |
| **Easy to Understand** | 311 (25) | Participants described PPG delivering comprehensible information through a platform they were already familiar with. They also describe PPG as making primary research results more accessible to a wide audience. | *“Easy to access, read & share. Easy to verify accuracy & reputation of information. I liked just seeing things in my feed. Sometimes I would come across information I'd file away for later.” – P8* |
|  |  |  | *“Backed information by science that is proven and well researched with thought and care for this community so we can make smart choices for ourselves and our families! Love how accessible and easy the content is to read and digest” – P729* |
|  |  |  | *“Easily accessible information that I can trust, breaks down medical information so it is easily to understand and directly applicable to my daily life.” – P187* |
| **Run by Trusted Professionals** | 306  (24) | Importance of PPG being run by clinicians working with pregnant and postpartum patients during the pandemic.  Some also highlighted the importance of the professionals being women and mothers. | *“The piece [peace] of mind that professionals from a reliable hospital were sharing and discussing many of the concerns and worries that I was having. It was so important during a very isolating time to feel like there was a community out there looking out for us.” – P836* |
|  |  |  | *“An account created by doctors that is as credible as possible. It's packaged up and well formatted for busy people wanting to get the most important information.” – P35* |
|  |  |  | *“Backed by research, physician led, info during pandemic specific to pregnancy and young children which was severely lacking from government/other news outlets.” – P645* |
| **Content was Up to Date and/or Relevant** | 246 (19) | Participants noted that it was important to them that the content on PPG was up to date, or relevant to their concerns at the time. Several participants mentioned that PPG was one of their “go-to” resources because they could often find information about new research before other sources, such as their doctors, knew about it. | *“I recommended it to all my own patients... it was a quick and easy way for me to stay up to date on the latest pregnancy related information related to covid. was an amazing supplement to other sources of information in my busy life!” – P973* |
|  |  |  | *“Canadian up to date content about babies and pregnancy. It is hard to find good up to date sources without retrieving academic articles yourself”* |
|  |  |  | *“It provided concise, digestible, up-to-date information. I felt confident sharing it with my friends and my healthcare providers. It also gave me a sense of community in an otherwise lonely time.” – P1011* |
| **Canadian or Locally Based** | 218 (17) | Participants noted that it was important to them that PPG was run by local physicians (their hospital, city, province or country) because they knew information about things that varied across jurisdictions (e.g., public health restrictions) was relevant for them. | *“Canadian content from a trusted Toronto hospital. I gave birth June 2020. And so much of the practical information required during covid (can I bring my partner to the hospital, will there be food in the hospital) was US based and unhelpful for parents in Canada with a different political landscape, healthcare and resources re: covid… Canadian content, that is empirically based - this info became my guide and gold standard during the pandemic, I saw studies on Instagram before my own doctor knew about them!” – P1950* |
|  |  |  | *“Canadian reliable physician. Initially I found it easier to access content created by American physicians. I felt that PPG filled a void I the communication we were receiving here. Particularly regarding the approval process and guidelines for the Canadian roll kit of vaccines. Enjoyed the live presentations” – P2061* |
|  |  |  | *“Science based content from Canadian physicians and mothers. Depicts relevant topics. You really helped me feel comfortable with getting vaccinated while pregnant, and it also helped me feel not so alone when I was very torn on that decision.” – P783* |
| **Easy to Access** | (n=136, 11%) | Participants described PPG as easy to access, given their familiarity with the  platform, as well as making primary research results  accessible to a wide audience | *“Easy to access, read & share. Easy to verify accuracy & reputation of information. I liked just seeing things in my feed. Sometimes I would come across information I'd file away for later.” – P8* |
|  |  |  | *“Easily accessible and reputable information that can be shared with friends and family when the internet is overflowing with mis/disinformation” – P1091* |
|  |  |  | *“Easily accessible anywhere, anytime run by health care professionals in women's and infants’ health Canada-based up to date sense of community at the height of COVID reliable information that are still applicable years after I initially joined (height of COVID)” – P2174* |
| **Provided Pregnancy, Postpartum, and Children’s Health Information** | (n=101, 8%) | The importance of having a resource that provided information specific to pregnancy, postpartum, children and women’s health during the pandemic. | *“Backed by research, physician led, info during pandemic specific to pregnancy and young children which was severely lacking from government/other news outlets.” – P645* |
|  |  |  | *“Easy to understand, timely information about COVID topics/recommendations/vaccination options available, and information about postpartum maternal mental health. PPG was often the first place I saw information reported about COVID related issues so I always felt in the loop and up to date on what resources were available (I.e. when vaccines and boosters would become/were available for myself and my child, etc.).” – P302* |
|  |  |  | *“I felt like PPG gave pregnant people a life raft during the pandemic. I felt like this helped fill the gap that I needed because I felt badly that my OB and GP were overrun with questions and stretched to their capacity. I love that PPG is succinct, clear, and presents information with a balanced approach. Feeling like I had an up-to-date, reliable, resource that was available to me during an incredibly stressful time, was imperative in helping me mediate my anxiety during my pregnancy and postpartum. The information on PPG made me feel confident in advocating for an early vaccination while pregnant. I got vaccinated at the earliest possible opportunity - 2 weeks prior to my delivery date.” – P95* |
| **Community Support** | (n=83, 7%) | Appreciating the support and tone (“feel”) of the PPG page, which they described as *open*, *honest, non-judgmental, and compassionate.*  Some also appreciated the support from knowing other followers were in similar situations. | *“Those were dark, worrisome and lonely times and during my maternity leave I had way too much time on my phone. I found the content trustworthy and informative, but also approachable, supportive and reassuring.” – P1813* |
|  |  |  | *“Trustworthy and backed by articles I can follow up on. Felt like a community. A place to ask questions when health care systems were overburdened.” – P1424* |
|  |  |  | *“While pregnant at the height of covid I relied heavily on the information and community that PPG gave me. I felt an overwhelming sense of calm and reassurance hearing from real Canadian doctors when I could barely get through to my own physician. I would prioritize watching live videos to hear their latest takes on everything covid and vaccinations while pregnant. I recommended following PPG and shared posts with my family and friends. PPG made me feel confident in my decision to get vaccinated while pregnant and encourage my partner to as well. I am so grateful for the invaluable guidance and support the PPG community and doctors gave me personally at such an uncertain time. Thank you to everyone who contributed!” – P584* |
